# N7-methylguanosine methylation of tRNAs regulates survival to stress in cancer

**DOI:** 10.1038/s41388-023-02825-0

**Published:** 2023-09-02

**Authors:** Raquel García-Vílchez, Ana M. Añazco-Guenkova, Judith López, Sabine Dietmann, Mercedes Tomé, Sonia Jimeno, Mikel Azkargorta, Félix Elortza, Laura Bárcena, Monika Gonzalez-Lopez, Ana M. Aransay, Manuel A. Sánchez-Martín, Pablo Huertas, Raúl V. Durán, Sandra Blanco

**Affiliations:** 1 Molecular Mechanisms Program, Centro de Investigación del Cáncer and Instituto de Biología Molecular y Celular del Cáncer, Consejo Superior de Investigaciones Científicas (CSIC)-University of Salamanca, 37007 Salamanca, Spain; 2 Instituto de Investigación Biomédica de Salamanca (IBSAL), Hospital Universitario de Salamanca, 37007 Salamanca, Spain; 3 Washington University School of Medicine in St. Louis, 660S. Euclid Ave, St. Louis, MO 63110 USA; 4 Centro Andaluz de Biología Molecular y Medicina Regenerativa-CABIMER, Consejo Superior de Investigaciones Científicas, Universidad de Sevilla, Universidad Pablo de Olavide, Sevilla, Spain; 5 Departamento de Genética, Universidad de Sevilla, Sevilla, Spain; 6 CIC bioGUNE, Basque Research and Technology Alliance (BRTA), Bizkaia Technology Park, 801 bld., 48160 Derio, Bizkaia Spain; 7 Carlos III Networked Proteomics Platform (ProteoRed-ISCIII), Madrid, Spain; 8 Centro de Investigación Biomédica en Red de Enfermedades Hepáticas y Digestivas (CIBERehd), Madrid, Spain; 9 Servicio de Transgénesis, Nucleus, Universidad de Salamanca, 37007 Salamanca, Spain

**Keywords:** Cancer therapeutic resistance, Transcriptomics

## Abstract

Tumour progression and therapy tolerance are highly regulated and complex processes largely dependent on the plasticity of cancer cells and their capacity to respond to stress. The higher plasticity of cancer cells highlights the need for identifying targetable molecular pathways that challenge cancer cell survival. Here, we show that N^7^-guanosine methylation (m^7^G) of tRNAs, mediated by METTL1, regulates survival to stress conditions in cancer cells. Mechanistically, we find that m^7^G in tRNAs protects them from stress-induced cleavage and processing into 5’ tRNA fragments. Our analyses reveal that the loss of tRNA m^7^G methylation activates stress response pathways, sensitising cancer cells to stress. Furthermore, we find that the loss of *METTL1* reduces tumour growth and increases cytotoxic stress in vivo. Our study uncovers the role of m^7^G methylation of tRNAs in stress responses and highlights the potential of targeting METTL1 to sensitise cancer cells to chemotherapy.

## Introduction

The ability of cancer cells to survive stress is crucial for tumour to progress and adapt to therapy, emphasising the need to identify targetable molecular pathways challenging cancer cell survival. RNA chemical modifications are emerging as an additional layer of biological information that dynamically regulate cellular functions, including self-renewal, proliferation, or survival to stress [1, 2]. Although the study of RNA modifications dates back to the 1950s, investigations into RNA modifications in the context of human cancer, began only around the 1990s. This focused exploration of RNA modifications in human cancer has revealed valuable insights into their role in tumorigenesis [3–9]. Understanding the specific involvement of RNA modifications in human cancer holds great promise for unravelling novel molecular mechanisms and identifying potential targets for diagnosis and treatment.

Over 170 RNA chemical modifications constitute the epitranscriptome, and their function is currently being investigated [10]. RNA modifications are primarily found in transfer RNA (tRNAs), where they regulate tRNA stability, folding, and protein synthesis [11]. Dysregulation of tRNA-modifying enzymes is frequently associated with stress-related diseases, including cancer [12]. For instance, reduced expression of TYW2 enhances migration in colorectal cancer [13]. Furthermore, altered tRNA modifications are implicated in cancer chemoresistance [14–17]. Notably, loss of tRNA cytosine-5 methylation (m^5^C) leads to increased production of stress-induced tRNA fragments (tRFs), sensitising cancer cells to stress and chemotherapy [18, 19]. These observations underscore the critical role of tRNAs and tRNA modifications in stress response, suggesting a potential link between tRNA modifications and therapy resistance. However, the precise molecular mechanisms that connect the stress response pathway to tRNA modifications remain largely unknown.

N^7^-methylguanosine (m^7^G) is one of the most common tRNA modifications in eukaryotes [20–22]. Its deposition is catalysed by the methylase complex formed by Methyltransferase 1 (METTL1), the catalytic subunit, and WD Repeat Domain 4 (WDR4), the regulatory subunit [20]. Increased expression of *METTL1* has been associated with various types of cancer [21–29], and chemotherapy sensitivity [15, 26, 30]. However, the precise molecular mechanisms connecting m^7^G modification and cellular stress response remain unclear.

Here, through transcriptome-wide approaches to map m^7^G, functional assays, and *Mettl1* knockout mouse models, we provide compelling evidence that m^7^G is essential for protecting tRNAs from stress-induced cleavage and protein synthesis regulation. Depletion of *METTL1* sensitises cancer cells to stress, resulting in heightened cytotoxic responses to conventional cancer treatments both in vitro and in vivo. Our study unveils the significance of m^7^G methylation of tRNAs in stress responses and highlights the potential of targeting METTL1 to enhance the sensitivity of cancer cells to therapy.

## Results

### Loss of N^7^-methylguanosine in tRNAs increases stress-induced tRNA cleavage

Hypomodified tRNAs have been linked with stress responses and chemosensitivity to targeted therapies [12, 14–17]. Because tumour cell tRNAs have been shown to be methylated by METTL1 [20, 31, 32], we postulate a potential connection between the loss of m^7^G deposition and stress signalling.

To validate the methylation of tRNAs by METTL1 in prostate cancer (PCa) cells (PC3), we developed a high-throughput method that precisely maps m^7^G deposition in RNA. This method utilises the sensitivity of methylated guanosines to chemical treatments, followed by RNA sequencing (RNA-seq) (Supplementary Fig. 1D) [33]. The absence of METTL1-mediated m^7^G methylation was confirmed in *METTL1* knock-out (*METTL1-KO*) cells (Supplementary Fig. S1A–C). RNA-seq was performed on treated and untreated RNAs (Supplementary Fig. S1D), and the cleavage sites in the treated RNAs served as a surrogate for m^7^G deposition sites.

**Fig. 1:**
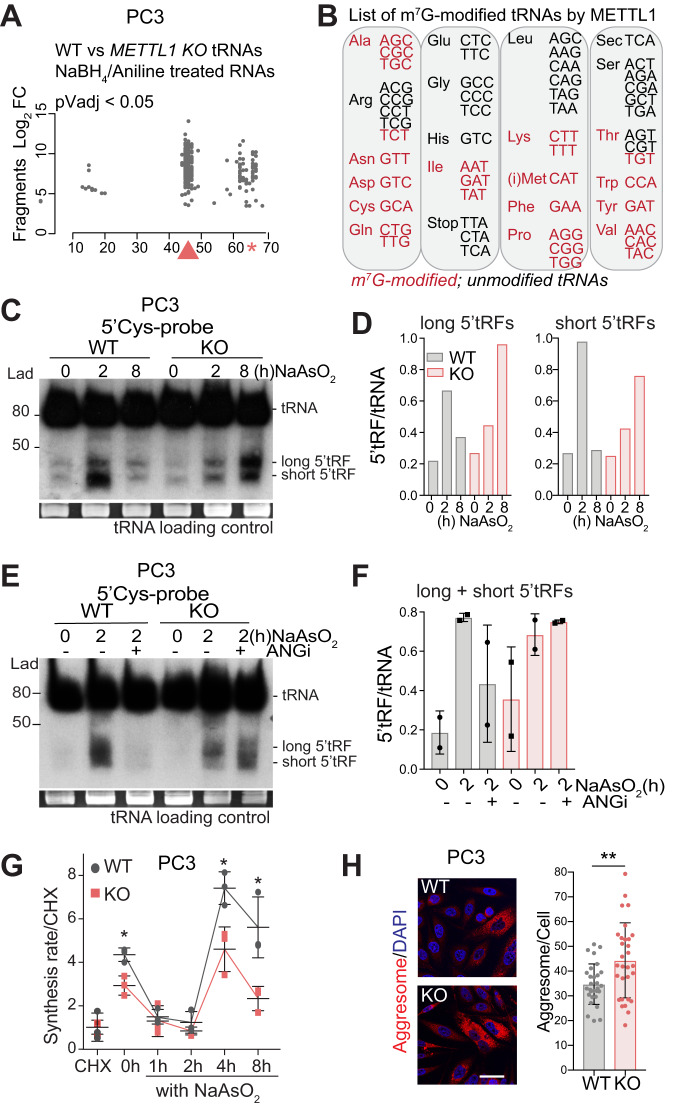
METTL1-mediated methylation protects tRNAs from stress-induced cleavage. **A** Mapping of METTL1-mediated guanosine-7 methylation in tRNAs of PC3-WT and *METTL1-KO* cells using NaBH_4_/Aniline-induced fragmentation analysis. The start position of statistically significant fragments formed in WT vs. *METTL1-KO* RNAs after NaBH_4_-treatment is indicated by grey dots. The red arrow indicates METTL1-methylated guanosines, the red star represents methylated guanosines of ArgTCT, IleTAT or TyrGTA which contain longer introns and variable loops, and hence position G46 corresponds to later positions that nucleotide 46. **B** Graphical summary showing tRNA isoacceptors methylated by METTL1 (red), and non-methylated or non-transcribed (black). **C**, **D** Detection and quantification of full-length Cys tRNA (upper bands) and 5’tRNA derived fragments (tRFs) using Northern blot analysis in PC3-WT and *METTL1-KO* cells under unexposed conditions (0 h) or exposed to oxidative stress (NaAsO_2_) for 2 and 8 h. Two types of 5’tRNA fragments (5’tRFs), long and short, were detected and quantified separately in **D**. Quantification was further normalised to full-length tRNAs. Detection (**E**) and quantification (**F**) of full-length Cys tRNA and 5’tRFs using Northern blot analysis in PC3-WT and *METTL1-KO* cells under unexposed conditions (0 h) or exposed to NaAsO_2_ for 2 h in the presence (+) or absence (-) of 96 μM of angiogenin inhibitor N65828 (ANGi). Long and short 5’tRFs were quantified together in **F**. Mean ± SEM, *n* = 2. Total tRNAs were stained with Red safe (**C**, **E**). **G** Protein synthesis rate in PC3-WT and *METTL1-KO* cells under unexposed conditions (0 h) or after exposure to NaAsO_2_, normalised with cycloheximide (CHX)-treated cells. Mean ± SD, *n* = 3. **H** Immunofluorescence of protein aggresomes and CTCF quantification in PC3-WT and *METTL1-KO* cells (left panel). Scale bar: 50 μm. Mean ± SD, *n* = 3. Stats: one-tailed Student’s *t*-test (**F**, **G**, **H**), ns: non-significative, *p < 0.05, **p < 0.01.

RNA-seq data analysis revealed increased cleavage of tRNAs at position G46 of the variable loop in approximately 50% of all tRNAs in PC3, including tRNAs PheGAA and AlaAGC (Fig. 1A, B, Supplementary Fig. S1E, F), and similar to other studies [19–21, 34]. tRNAs from *METTL1-KO* cells were not cleaved at position G46, confirming the loss of m^7^G deposition (Supplementary Fig. S1E, F, Supplementary Table S1).

Because loss of m^5^C at the variable loop of tRNAs has been associated with an increased generation of stress-induced 5’tRFs processed by angiogenin [14, 18, 19, 34], our next objective was to determine whether the loss of m^7^G could similarly impact on tRNA processing. We found no significant differences in the stability of mature tRNA in PC3 cells lacking *METTL1* (Supplementary Fig. S1G). Subsequently, we examined the generation of oxidative stress-induced 5′tRFs. As expected, tRNAs were cleaved in PC3 WT cells (Fig. 1C, D), resulting in two different 5′tRFs: longer fragments presumably corresponding to 5′tRNA halves cleaved at the anticodon loop, and shorter fragments presumably cleaved at the D loop. Fragment production peaked at 2 h after stress stimulation and disappeared at 8 h, in accordance with previous reports [14]. Interestingly, the production of tRFs in *METTL1-KO* cells was higher at later points, suggesting a prolonged stress response (Fig. 1C, D). Furthermore, tRNA cleavage in PC3 *METTL1-KO* cells was not suppressed in the presence of the angiogenin inhibitor, indicating the involvement of another endonuclease in tRNA cleavage (Fig. 1E, F, Supplementary Fig. S1H).

Thus, our data demonstrate that the biogenesis of distinct stress-induced 5’tRFs is driven by METTL1-specific methylation.

### Loss of N^7^-methylguanosine in tRNAs disrupts proteostasis

Stress-mediated tRNA cleavage and 5′tRFs are important components of the cellular stress response that ultimately inhibits global protein synthesis to allow cells to recover from stress and survive [35]. Accordingly, analysis of protein synthesis rate in PC3 *METTL1-KO* cells showed a significative reduction in protein translation and a weakened recovery of protein synthesis rates compared to WT cells (Fig. 1G, Supplementary Fig. S1I). These findings were validated in the PCa cell line DU145 using doxycycline-inducible shRNAs against *METTL1*. Consistently, the immediate loss of m^7^G deposition in tRNAs led to a consistent decrease in protein synthesis (Supplementary Fig. S1J, K). These data strongly indicated that loss of m^7^G caused stress-induced repression of protein synthesis across different cell lines. To further asses proteostatic stress in the absence of *METTL1*, we examined the formation of protein aggregates. *METTL1*-deficiency led to a significant increase in protein aggregates formation, confirming an unbalanced protein homoeostasis (Fig. 1H).

In summary, we conclude that loss of METTL1-mediated tRNA methylation disturbs tRNA processing and leads to unbalanced proteostasis.

### Loss of *METTL1* disrupts the autophagic flux

**Fig. 2:**
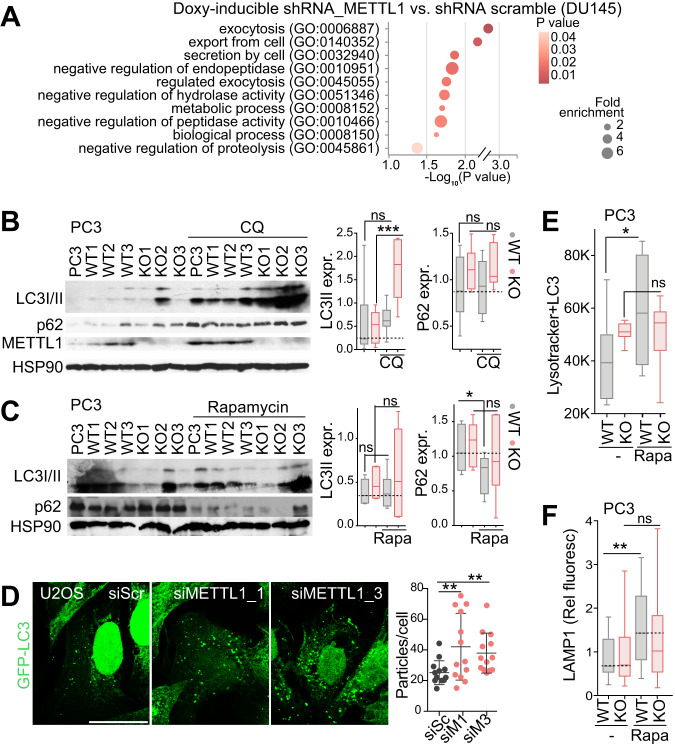
Impaired autophagy resolution in the absence of METTL1. **A** Gene Ontology (GO) category enrichment analysis of statistically significant dysregulated proteins identified through quantitative proteomics in *METTL1*-silenced DU145 cells using doxycycline-inducible shRNAs. **B**, **C** Western blot analysis (left) and protein quantification (right) of LC3 I/II, p62, and METTL1 in three clones of PC3-WT and *METTL1-KO* cells cultured in complete glucose, amino acid and serum medium, without or with chloroquine (CQ) treatment (**B**) or rapamycin (Rapa) treatment (**C**). Mean±Max-Min, *n* = 6. **D** Immunofluorescence analysis (left) and quantification (right) of GFP-LC3 puncta in *METTL1-*silenced U2OS cells using siRNA. Control cells were transfected with a scramble (SCR) siRNA. Mean±Max-Min, *n* = 3 images. Scale bar: 25 μm. **E** Number of PC3-WT and *METTL1-KO* cells double positive for Lysotracker and LC3, cultured in complete medium alone (-) or under rapamycin treatment. Mean± Max-Min, *n* = 3. **F** Expression levels of endogenous LAMP1 in PC3-WT and *METTL1-KO* cells without (-) or with rapamycin treatment. Mean±Max-Min, *n* = 9. Stats: one-tailed Student’s *t*-test (**B**–**F**). *p < 0.05, **p < 0.01, ***p < 0.001.

Since tRNA modifications and tRFs can modulate translational programmes, we next used proteome-wide profiling of *METTL1*-inducibly silenced DU145 cells to specifically analyse the direct proteome changes caused upon the removal of *METTL1*. We identified subtle expression changes in common proteins upon *METTL1*-silencing (Supplementary Table S2). GO term analysis revealed enrichment in catabolic processes such as proteolysis, peptidase and hydrolase activities (Fig. 2A), suggesting a potential link between METTL1 and autophagy in line with previous findings [36]. To explore this, we examined the levels of LC3 II and p62 in PC3-WT and *METTL1-KO* cells. No significant expression changes were found in *METTL1-KO* cells compared to WT cells growing in culture conditions with complete or reduced medium with amino acids and serum (Fig. 2B, C, Supplementary Fig. S2A–C). However, *METTL1* depletion in PC3 cells significantly increased the levels of LC3 II after chloroquine (CQ) treatment, which inhibits the latest steps of autophagy, indicating that the loss of *METTL1* enhanced autophagosome formation (Fig. 2B, Supplementary Fig. S2A). p62 levels remained unchanged after CQ treatment, most likely due to reduced autophagosome resolution (Fig. 2B, Supplementary Fig. S2A). Amino acids and serum starvation in PC3 *METTL1-KO* cells led to higher LC3 II accumulation, which was further increased by CQ, suggesting impaired fusion of autophagosomes with lysosomes in these cells (Supplementary Fig. S2C). Similar effects were observed in DU145 *METTL1-KO* cells (Supplementary Fig. S2D, E). p62 levels were scarcely changed after 4 h of starvation in PC3 and DU145 *METTL1-KO* cells (Fig. 2C, Supplementary Fig. S2B, C, D, E). However, autophagy induction with rapamycin treatment significantly reduced p62 levels in WT cells but failed to stimulate p62 degradation in *METTL1-KO* cells, indicating that METTL1 is necessary for autolysosome maturation (Fig. 2C, Supplementary Fig. S2B).

To confirm impaired autolysosome maturation in *METTL1*-deficient cells, we utilised U2OS cells expressing the autophagosome reporter LC3-GFP. Silencing of *METTL1* using siRNAs induced an increased accumulation of GFP+ puncta, confirming an increased formation of autophagosome vesicles (Fig. 2D, Supplementary Fig. S2F). Additionally, PC3-WT and *METTL1-KO* cells were transfected with LC3-GFP reporter plasmid and accumulation of LC3-GFP + /lysotracker+ or LAMP1 + -vesicles was measured. In PC3-WT cells, rapamycin induction resulted in accumulation of autolysosomes, indicating an increased autophagic flux. However, in *METTL1-KO* cells, the formation of autolysosomes did not change compared to rapamycin-untreated cells, suggesting reduced autophagic flux (Fig. 2E, F, Supplementary Fig. S2G, H, I). Thus, our data indicated that *METTL1* loss disrupted the autophagic flux by impairing or reducing autophagy resolution. Although Han et al. [26] observed a decreased translation of mTOR and other negative autophagy regulators in oesophagus squamous cell carcinoma, we did not find significant or consistent changes in phosphorylation of downstream effectors of mTORC1 in PCa cell lines (Supplementary Fig. S2J).

Together, our data revealed that METTL1 is necessary to resolve autophagy independent of mTOR status, its loss induces unresolved autophagy and increased proteostatic stress.

### Inhibiting the expression of *METTL1* leads to a decrease in cell viability

**Fig. 3:**
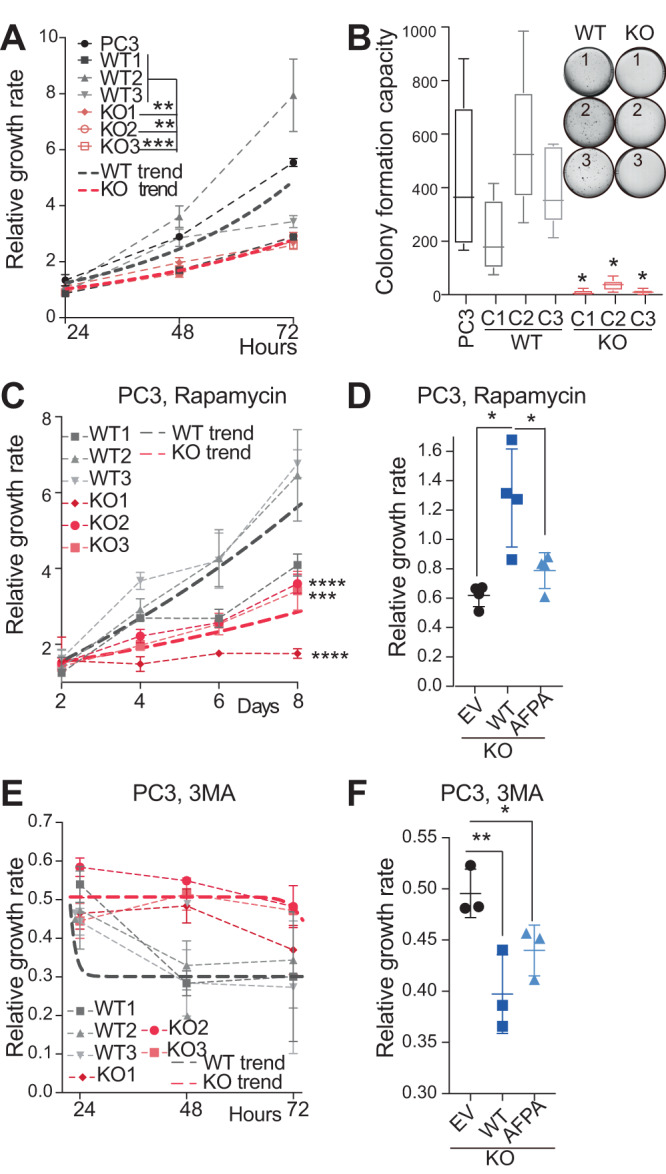
Decreased cell viability upon METTL1 inhibition. **A** Proliferation of three clones of PC3-WT and *METTL1-KO* cells. Thicker dotted lines represent the average growth of all WT or *METTL1-KO* clone-derived cell lines. Mean ± SD, *n* = 5. **B** Soft-agar assay of PC3-WT and *METTL1-KO* cells. **C**, **E** Representative images of soft-agar colonies are shown in the top right panel. Mean±Min-Max, *n* = 6. Proliferation of three clones of PC3-WT and *METTL1-KO* cells treated with the autophagy inducer rapamycin (**C**) or the autophagy inhibitor 3MA (**E**). Mean ± SD, *n* ≥ 3. The thicker dotted lines represent the average growth of all WT or *METTL1-KO* clone-derived cell lines. **D**, **F** Relative growth of PC3 *METTL1-KO* cells expressing an empty vector (EV), HA-METTL1 (WT) or the catalytic dead mutant AFPA, treated with rapamycin (**D**) or 3MA (**F**). Growth under doxycycline-induced versus non-induced conditions is shown. Mean ± SD, *n* = 4 (**D**), *n* = 3 (**F**). Stats: two-way ANOVA (**A**, **B**, **C**, **E**), one-tailed Student’s *t*-test (**D**, **F**). *p < 0.05, **p < 0.01, ***p < 0.001, ****p < 0.0001.

tRNA modifications and tRFs play a critical role in cell viability and survival under stress conditions and can act as stress sensors [14, 18, 19, 35, 37, 38]. Taken together, these findings suggest that the loss of *METTL1* could reduce cell viability. Indeed, our functional analysis demonstrated that the loss or knockdown of *METTL1* in PCa cells decreased cell proliferation and reduced 3D growth (Fig. 3A, B, Supplementary Fig. S3A). Moreover, *METTL1*-deficient cells exhibited increased cell sensitivity to oxidative stress as shown by a decrease in IC_50_ to H_2_O_2_ (Supplementary Fig. S3B). Consequently, *METTL1* loss resulted in decreased cell viability in response to oxidative stress or UV exposure (Supplementary Fig. S3C, D).

Considering that autophagy resolution was disrupted in *METTL1*-deficient cells, we investigated whether autophagy induction could further compromise cell survival. As expected, PC3 *METTL1-KO* cells exhibited increased sensitivity to rapamycin compared to WT cells (Fig. 3C, Supplementary Fig. S3E), likely due to the accumulation of unresolved autophagosomes. The survival defects observed in PC3 *METTL1-KO* cells were rescued upon re-expression of a wild-type METTL1 form, but not by a catalytically inactive version (AFPA), indicating that tRNA methylation is necessary for survival regulation (Fig. 3D, Supplementary Fig. S3F). Interestingly, PC3 *METTL1-KO* cells exhibited higher tolerance than WT cells to 3-methyladenine (3MA), which blocks autophagosome formation by inhibiting class III PI3K [39]. This suggested that reducing autophagy initiation could partially restore *METTL1-KO* cells viability (Fig. 3E). The re-expression of wild-type METTL1 significantly reduced cell viability, while the METTL1 inactive mutant partially reduced cell viability (Fig. 3F).

In conclusion, our data highlight the essential role of METTL1-mediated tRNA methylation in maintaining cell viability and promoting survival under stress conditions, supporting its oncogenic role reported in recent studies [21–29, 40–42].

### *METTL1* inhibition sensitises cells to genotoxic stress

**Fig. 4:**
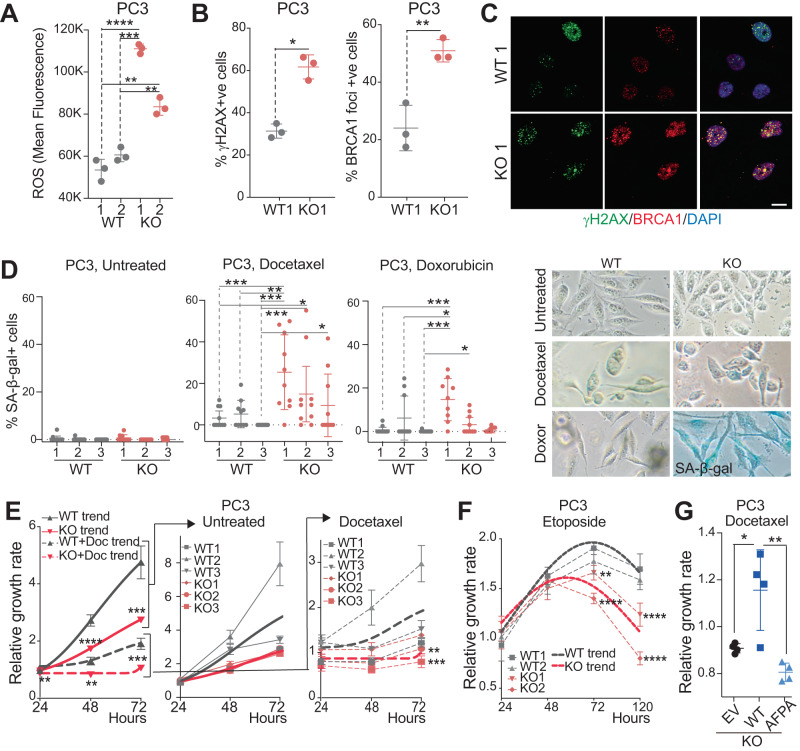
Induction of DNA damage and senescence in *METTL1-KO* cells. **A** Intracellular Reactive Oxygen Species (ROS) levels in two clones of PC3-WT and *METTL1-KO* cells. Mean ± SD, *n* = 3. Quantification (**B**) and immunofluorescence (**C**) of γH2AX- and BRCA1-foci positive cells as a measure of DNA damage in PC3-WT and *METTL1-KO* cells. Mean ± SD, *n* = 3, and 100 cells analysed per replicate. Scale bar indicates 20 μm. **D** Senescence-associated β-galactosidase activity (in blue) in untreated and treated cells with Docetaxel, and Doxorubicin for three days. Three clones of PC3-WT and *METTL1-KO* cells are shown. Representative images of β-gal+ cells are sown in right panels. Mean ± SD, *n* = 3, 10 images each replicate. Viability of PC3-WT and *METTL1-KO* cells untreated or treated with Docetaxel (**E**) or Etoposide (**F**). Thicker dotted lines represent the average growth of three clones of WT or *METTL1-KO* clone-derived cell lines. Mean ± SEM, *n* = 6. **G** Viability of PC3 *METTL1-KO* cells ectopically expressing a doxycycline-inducible empty vector (EV), HA-METTL1 (WT) or a catalytic dead mutant (AFPA) and treated with Docetaxel. Stats: one-tailed Student’s *t*-test (**A**, **B**, **D**, **G**), two-way ANOVA (**E**, **F**). *p < 0.05, **p < 0.01, ***p < 0.001, ****p < 0.0001.

Disruption of autophagy frequently leads to the accumulation of reactive oxygen species (ROS), which is often associated with heightened genotoxic stress [43, 44]. We next examined whether the dysregulation of autophagy in the absence of *METTL1* affected the cellular redox balance. We detected higher ROS production in PC3 *METTL1-KO* than in WT cells (Fig. 4A, Supplementary Fig. S4A). Furthermore, *METTL1* deletion significantly increased the formation of γH2AX nuclear foci, indicative of DNA double-strand breaks, accompanied by a significant increase in the formation of BRCA1 foci near the sites of DNA damage [45] (Fig. 4B, C, Supplementary Fig. S4B, C).

Loss of autophagy is often linked to cellular senescence [46] triggered by DNA damage [47]. Under normal conditions, *METTL1* depletion did not reveal a significant increase in senescence induction (Fig. 4D). However, upon Docetaxel or Doxorubicin treatment [48], a significant increase of senescence was observed in PC3 *METTL1-KO* cells (Fig. 4D). Similarly, *METTL1* silencing in 22Rv1 cells, which express p16 and p53 (Supplementary Fig. S4E), significantly induced senescence (Supplementary Fig. S4F). We also analysed the acquisition of a senescence-associated secretory phenotype (SASP), characterised by the secretion of inflammatory cytokines, growth factors, and matrix metalloproteinases [49]. The expression of common SASP factors was found upregulated in *METTL1-*deficcient PC3 cells (p53-deficient), DU145 cells (p53-WT), and upon *METTL1*-silencing in 22Rv1 cells (p53-WT) (Supplementary Fig. S4D, G, H).

Because autophagy and senescence function as survival mechanisms to resolve stress conditions [44, 48], we investigated whether inhibiting METTL1 would sensitise cells to chemotherapy-induced stress. *METTL1* deletion in PC3 cells increased cellular sensitivity to chemotherapeutic agents like Docetaxel and Etoposide (Fig. 4E, F). Re-expression of wild-type METTL1, but not the catalytically inactive mutant AFPA, in PC3 *METTL1-KO* cells reduced their sensitivity to genotoxic stress (Fig. 4G), highlighting the importance of METTL1-dependent methylation in cellular stress responses.

In summary, our findings suggest that inhibiting METTL1 alone or in combination with chemotherapeutic agents could enhance the elimination of cancer cells within a tumour by rendering them more susceptible to genotoxic stress and increasing their responsiveness to chemotherapy.

### *METTL1* is highly expressed in luminal cells in PCa

**Fig. 5:**
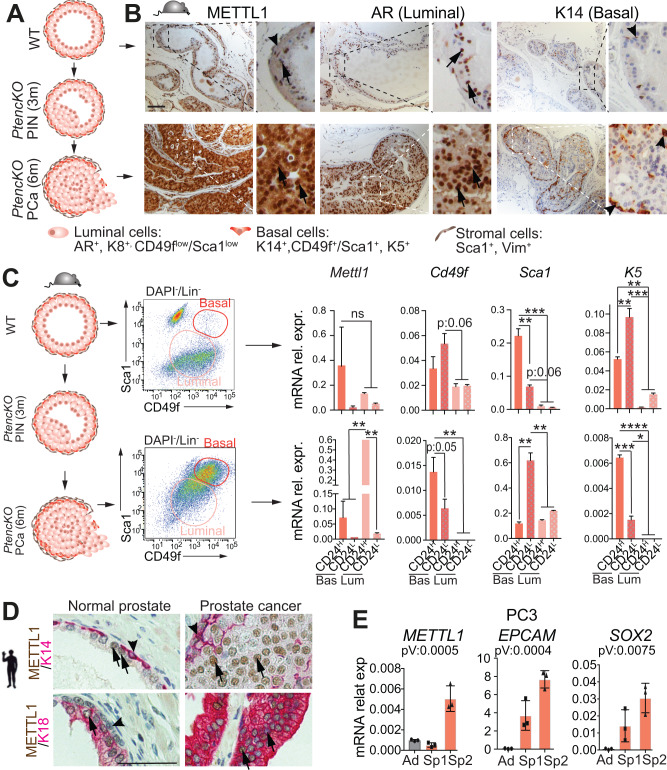
High expression of *METTL1* in PCa. **A** Schematic overview of PCa progression in *PtencKO*-mice: mice developed prostatic intraepithelial neoplasia (PIN) at 3 months (3 m), and prostate invasive carcinoma (PCa) at 6 months (6 m). Cell types and expression markers used are shown at the bottom. **B** Immunostaining of METTL1, AR (luminal marker), and K14 (basal marker) in healthy prostates (WT) and prostate tumours (*PtencKO)*. Scale bar: 50 μm. **C** Prostate cells from healthy prostates (WT) and tumours (*PtencKO)* were sorted based on gates shown in middle panel plots and Supplementary Fig. S5A, and RNA was extracted. mRNA expression levels of *Mettl1* and cell markers are shown for basal (Bas) and luminal (Lum) cells with high (CD24^H^) or low CD24 (CD24^L^) expression. **D** Immunostaning of METTL1, K18 (luminal), and K14 (basal marker) in human normal prostate and PCa. Scale bar: 50 μm. **E** mRNA expression levels of *METTL1*, and the stem cell markers *EPCAM* and *SOX2* in PC3 cells growing in normal culture conditions (Ad) and in self-renewing conditions: spheroids at passage 1 and 2 (Sp1, Sp2). Mean ± SD, *n* = 3. Arrows indicate luminal cells. Arrowheads indicate basal cells (**B**, **D**). Stats: one-way ANOVA test (**C**, **E**). *p* < 0.05, **p < 0.01, ***p < 0.001, ****p < 0.0001.

To investigate the potential significance of METTL1-mediated methylation in cancer cells, we examined *METTL1* expression in this population. *METTL1* has been found upregulated in several tumours [21–28, 40–42], yet, its expression in self-renewing cancer stem cells remains unknown. To address this, we utilised the PCa mouse model developed by Professor P.P. Pandolfi [50] carrying the Probasin-Cre/*Pten*^*flox/flox*^ genotype *(PtencKO)*, which displays high-grade pre-neoplastic lesions with complete penetrance within three months, which progress to invasive adenocarcinoma by six months (Fig. 5A) [50]. Prostate invasive adenocarcinomas exhibited an increase in Mettl1+ cells and elevated *Mettl1* expression compared to healthy tissue (Fig. 5B). Moreover, those *Mettl1*-expressing cells were predominantly luminal cells, similar to cells expressing androgen receptor (AR). Analysis of fluorescence-activated cell sorting (FACS)-sorted cells confirmed higher *Mettl1* expression in Sca1^low^/CD49f^low^/CD24^high^ luminal cells within prostate tumours (Fig. 5C, Supplementary Fig. S5A), which have been identified as the preferred cell-of-origin in most lineage-tracing and organoid studies [51–55].

Immunohistochemical analysis confirmed high expression of *METTL1* in luminal (K18 +) cells in human prostatic carcinoma samples (Fig. 5D). To further investigate *METTL1* expression in self-renewing cells of human cancer, we employed an in vitro model utilising formation by cancer stem cells. Tumoursphere cultures derived from PC3 and DU145 cells exhibited higher expression of *METTL1*, along with the stem-cell markers, compared to cells in 2D-growing conditions, confirming higher expression of *METTL1* in self-renewing human PCa cells (Fig. 5E, Supplementary Fig. S5B).

In summary, our findings indicate that METTL1 is prominently expressed in luminal cells, which are frequently identified as the cell of origin in mouse and human PCa.

### Inhibition of *Mettl1* expression sensitises cancer cells to chemotherapy in vivo

**Fig. 6:**
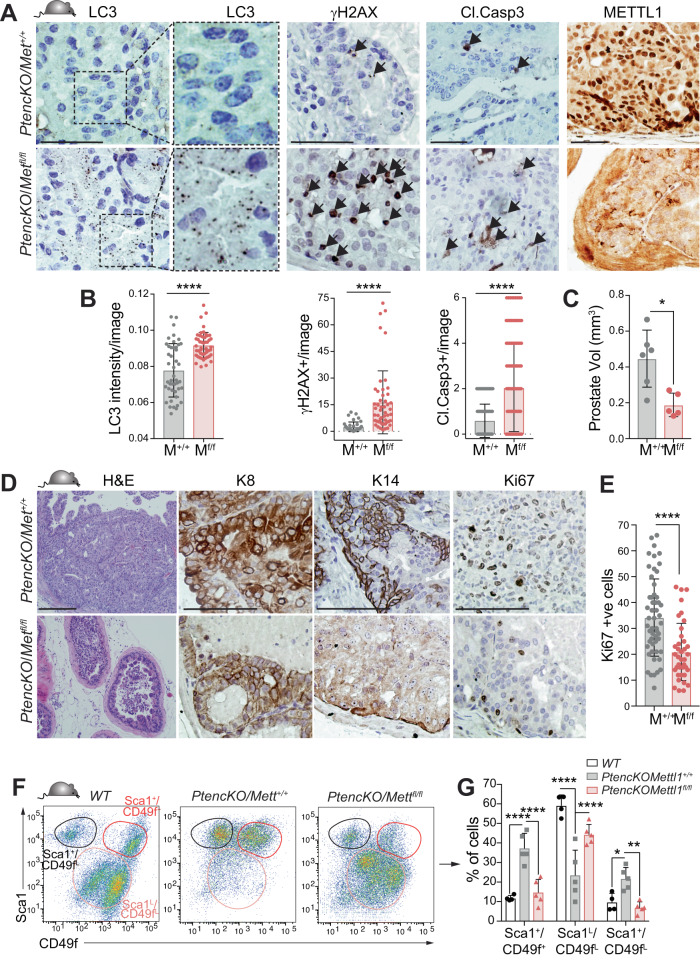
*Mettl1* inhibition disrupts autophagy and decreases viability in PCa cells in vivo. Immunostained images (**A**) and quantification (**B**) of LC3 levels, γH2AX+ cells, cleaved-Caspase 3+ (Cl.Casp3) cells, in PCa from *PtencKO*/Met^+/+^ and *PtencKO*/Met^fl/fl^ mice. Arrows indicate positive cells. Mean ± SD, *n* = 4 mice, 10 images per mouse. **C** Prostate tumour volumes of *PtencKO*/Met^+/+^ (M^+/+^) and *PtencKO*/Met^fl/fl^ (M^fl/fl^) mice. Mean ± SD, *n* ≥ 5. **D** Immunostained images of markers of luminal (K8), and basal cells (K14), proliferation (Ki67), and Hematoxylin and Eosin staining of tumours from *PtencKO*/Met^+/+^ and *PtencKO*/Met^fl/fl^ mice. **E** Quantification of Ki67+ cells in mouse PCa. Mean ± SD, *n* = 4 mice, 10 images per mouse. Cell population distributions analysed by flow cytometry (**F**) and quantification (**G**) of prostatic cell subpopulations from healthy (WT), *PtencKO*/Met^+/+^ and *PtencKO*/Met^fl/fl^ mice according to cell surface markers Sca1 and CD49f. Mean ± SD, *n* = 5. Scale bar: 25 μm (**A**), 50 μm (**D**). Stats: Mann–Whitney test (**B**, **C**, **E**), Two-way ANOVA (**G**) *p < 0.05, **p < 0.01, ****p < 0.0001.

To investigate the impact of inhibiting Mettl1 in an in vivo setting, we conditionally deleted *Mettl1* in the prostatic epithelium of *PtencKO* mice (*PtencKO/Mettl1*^*flox/flox*^) (Fig. 6A, Supplementary Fig. S6A). Conditional *Mettl1* loss led to increased expression of autophagy and genotoxic stress markers in cancer cells (Fig. 6A, B). Accordingly, *Mettl1*-deficient tumours exhibited a significant increase in apoptosis (Fig. 6A, B), leading to a substantial reduction in tumour mass (Fig. 6C). Histological examination revealed that WT tumours were invasive, composed of proliferative (Ki67 +) luminal (K8 +) and basal (K14 +) cells. In contrast, *Mettl1*-deficient tumours were encapsulated and predominantly comprised of luminal cells with low proliferative potential (Fig. 6D, E). Furthermore, cytometry analyses indicated that depletion of *Mettl1* resulted in a cell subpopulation distribution similar to non-tumoural tissue (Fig. 6F, G). These data suggest that cancer cells and cancer stem-like cells are sensitive to the absence of this enzyme.

**Fig. 7:**
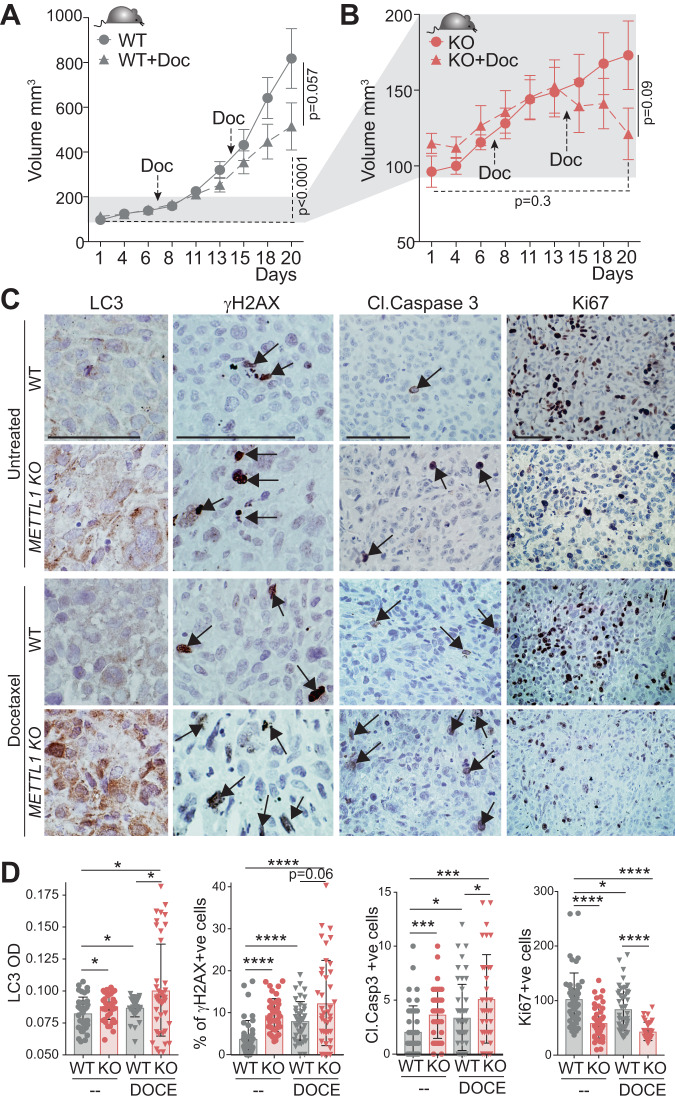
*METTL1* expression inhibition increases the efficiency of chemotherapy in PCa. Tumour growth of xenografted PC3-WT (**A**) and *METTL1-KO* cells (**B**) in athymic nude mice untreated or treated with Docetaxel. Arrows indicate days of treatment. Mean ± SEM, *n* = 9. Immunostained images (**C**) and quantification (**D**) of LC3 levels, γH2AX +, cleaved-Caspase 3 +, and Ki67+ cells in xenografts. Arrows indicate positive cells. Mean ± SEM, *n* = 4 mice, 10 images per mouse. Scale bar: 50 μm. Stats: Mann–Whitney test (**A**, **B**, **D**), *p < 0.05, **p < 0.01, ***p < 0.001, ****p < 0.0001.

To test whether the stress-activated programme in *Mettl1*^*-/-*^ cancer cells could alter their sensitivity to chemotherapy in vivo, we administered Docetaxel, commonly used for treating of metastatic castration-resistant prostate cancer (mCRPC) [56], to mice-bearing tumours. While Docetaxel treatment induced a modest growth reduction in wild-type tumours (Fig. 7A), it significantly reversed the growth and reduced the size of *METTL1-KO* tumours, almost to the initial stages of tumour formation (Fig. 7B). Docetaxel-treated *METTL1-KO* tumour cells exhibited dysregulated autophagy, increased DNA damage and apoptosis, leading to a reduction in cells re-entering the cell cycle (Ki67+cells) compared to wild-type cells (Fig. 7C, D). Similar results were obtained using another genotoxic agent, Etoposide (Supplementary Fig. S7). Thus, our data demonstrate that *METTL1*-deficient tumours fail to activate survival pathways in response to stress.

Taken together, our findings suggest that METTL1 plays a role in regulating cancer cell stress responses. Its inhibition results in increased sensitivity to stress, decreased cell viability, and reduced survival. Consequently, the inhibition of METTL1, alone or in combination with chemotherapeutic options, leads to a substantial reduction in tumour mass, resulting in tumours that resemble the architecture of healthy tissue.

## Discussion

Here, we demonstrate that METTL1, by methylating tRNAs, is vital for regulating cell viability and stress responses. Loss of METTL1-mediated 7-methylguanosine in tRNAs leads to the accumulation of stress-related small noncoding RNAs derived from 5’tRFs, disrupting proteostasis and catabolic cell states. Consequently, *METTL1*-deficient cells exhibit heightened sensitivity to stress stimuli and display imbalances in redox regulation, increased DNA damage, and senescence. Inhibiting *METTL1* expression in tumours impedes their progression and reverts them to a state resembling healthy tissue. Furthermore, METTL1 inhibition renders cancer cells more susceptible to the effects of chemotherapeutic agents, offering potential in combination therapy for enhanced effectiveness.

These benefits are particularly relevant in PCa, which ranks as the second most diagnosed cancer among men worldwide, and in patients diagnosed with mCRPC, the 5-year survival rate is 30%. For a long time, Docetaxel in combination with prednisone has been the primary therapeutic option for these patients. However, the heterogeneity of mCRPC necessitates the identification of new targetable molecular pathways to improve treatment outcomes [56, 57].

Targeting *METTL1* presents an attractive molecular approach as it is overexpressed in PCa. *METTL1* is also upregulated in other tumour types [21–29, 40–42], which highlights the potential of METTL1 inhibitors as novel therapeutic options in cancer. Moreover, *METTL1* expression levels could serve as a valuable biomarker to stratify patients as responders or non-responders to Docetaxel- or rapamycin-based therapies. Further investigations into the relationship between *METTL1* expression and response to chemotherapy will be valuable in refining personalised cancer therapeutics.

The development of targeted inhibitors for specific enzymes involved in RNA modifications holds great potential for therapeutic interventions in cancer. While there are currently no METTL1-specific inhibitors available, recent breakthroughs in the field of epitranscriptomics and cancer have led to the successful development of inhibitors targeting METTL3, an RNA 6-methyladenosine transferase [58]. These inhibitors have shown promising results in preclinical models of leukaemia, demonstrating the therapeutic value of targeting RNA modification enzymes. The successful development of METTL3 inhibitors provides a promising starting point for the design of inhibitors targeting other METTL members, including METTL1. The recent structural characterisation of the METTL1-WDR4 complex and the elucidation of the RNA recognition and methylation mechanism have further enhanced our understanding of METTL1’s function [59, 60], which provides valuable insights for the development of specific inhibitors.

Recent studies have also revealed that METTL1 inhibition is associated with increased sensitivity to chemotherapy, although the specific functions and molecular mechanisms of tRNA m^7^G methylation remain largely unknown [15, 21–28, 40, 42]. In our current research, we have discovered that METTL1 plays a crucial role in regulating the production of 5’tRFs, which trigger a stress response in cancer cells, ultimately leading to enhanced sensitivity to genotoxic agents. These stress-induced 5’tRFs are specifically generated to repress protein translation [14]. While the cellular factors governing tRNA cleavage are not yet well understood, recent studies have highlighted the importance of m^5^C, pseudouridine, and adenine methylation as critical determinants of tRNA fate under stress conditions [14, 61, 62]. For instance, NSUN2-mediated methylation of tRNAs regulates the generation of 5’tRFs, which in turn activate stress signalling, induce proteostatic stress, and ultimately decrease the functionality and stress sensitivity of stem and cancer stem cells [18, 19]. Therefore, our findings, combined with previous reports, indicate that maintaining tRNA integrity and tRNA modifications are essential for cell survival, and disrupting these processes in combination with current chemotherapeutic options could offer more effective strategies for eliminating cancer cells.

*METTL1* is highly expressed in various types of cancer, highlighting its significant role in tumorigenesis. However, it is still unclear whether METTL1 is involved in the survival of cancer cells [21–28, 40–42]. Only recent studies have suggested that targeting autophagy could be a promising approach for treating tumours with high *METTL1* expression [26]. Nevertheless, the precise mechanism by which METTL1 connects the regulation of catabolic pathways and the fitness of cancer cells remains poorly understood. In our study, we uncover that METTL1-mediated tRNA methylation plays a role in the survival and catabolic states of cells by posttranscriptionally regulating the generation of stress-associated 5’tRFs. Interestingly, the abnormal accumulation of these 5’tRFs has been linked to metabolic and neurological disorders, further emphasising their significance [63].

Our findings also demonstrate that METTL1 regulates stress pathways associated with proteostatic stress, autophagy, redox signalling, DNA damage, and senescence [44]. These pathways work together to enable cancer cells to adapt to severe stress conditions. Here, we report that *METTL1* depletion leads to autophagy inhibition, elevated ROS production, and DNA damage, suggesting that combining METTL1 inhibitors with chemotherapy can improve therapeutic responses in cancer.

Excessive DNA damage can induce both cell death or cell cycle arrest through senescence [64], which has both tumour-suppressive and drug-tolerant persistence characteristics [65, 66]. These issues can be overcome by combination therapies, since higher doses have been described to induce apoptosis of cells, which can be further eliminated by treatment with senolytic compounds [67]. This suggests a potential strategy for *METTL1*-overexpressing tumours, where the use of combination therapies, including METTL1 inhibitors, may synergise with chemotherapy or senolytic therapy to promote more effective elimination of tumour-initiating cells and persistent cancer cells.

In conclusion, our findings unveil a post-transcriptional mechanism for controlling essential functions in cancer cells and uncover novel therapeutic strategies to enhance tumour cell lethality.

## Materials and methods

### Cell culture

PC3, DU145, 22Rv1, and HEK293FT cells were from DSMZ and ThermoFisher Scientific. U2OS cells stably expressing GFP-LC3 were provided by R. V. Durán (CABIMER, Spain). PC3, DU145, U2OS, and HEK293FT were cultured in DMEM with L-Glutamine and pyruvate (Gibco). 22Rv1 was cultured in RPMI with L-Glutamine and pyruvate (Gibco). Media were supplemented with 10% Foetal Bovine Serum (FBS, Gibco) and 1% Penicillin/streptomycin. All cells were maintained at 37 °C and 5% CO_2_ and routinely tested for Mycoplasma.

### Stable cell line generation

HEK293FT cells were transfected with JetPEI (PolyPlus) and third-generation lentiviral packing vectors. After 48 h, viral particles were collected from the medium and added to cells with 8 μg/ml of protamine sulfate (Sigma-Aldrich). Selection was performed with 2 μg/ml puromycin (Sigma-Aldrich) or 10 μg/ml blasticidin (Santa Cruz) for 3–5 days.

PC3 and DU145 *METTL1-KO* cells were generated using CRISPR/Cas9 technology. Single-guide RNAs (sgRNAs) targeting exons 2 and 3 of *METTL1* were designed using *crispor* (http://crispor.tefor.net/) and cloned into pLentiCRISPR-v2 (Addgene, 83480). sgRNA used were: sg117-5’-TATGTCTGCAAACTCCACTTGGG-3’; sg139: 5’-CAAGTGGAGTTTGCAGACATAGG-3’). Control cells were transduced with LentiCRISPR-v2 expressing only Cas9 protein. Single-cell clones were generated for each sgRNAs and validated through sequencing, RT-qPCR, Western Blot or immunofluorescence.

For the generation of DU145 cells stably expressing doxycycline-inducible shRNAs for human *METTL1*, pLKO.1-TetON-puro vector (Addgene, 21915) was used. Control cells were transduced with Tet-pLKO.1-TetON-puro-scrambled (shRNA_scramble) (Addgene, #47541). shRNA sequences against *METTL1* were obtained from the Sigma Human Mission shRNA library (cat number: NM_005371): shMETTL1-3 (clone ID: TRCN0000331664) and shMETTL1-4 (clone ID: TRCN0000035958).

To generate PC3 *METTL1-KO* cells stably expressing inducible HA-METTL1 or HA-METTL1-AFPA, we cloned HA-METTL1 using the ORF from Source BioScience (clone: X96698; IMAGE clone: 36006) into doxycycline-inducible pTRIPZ (Dharmacon). For METTL1-AFPA generation, site-directed mutagenesis was performed using Q5 Site-Directed Mutagenesis Kit (NEB) and primers (F: 5′-CCCGCCCCACATTTCAAGCGGACA-3′ and R: 5′-GAAGGCGAAGAACATCTTTGTCAGC-3′). PC3, DU145, and PC3 *METTL1-KO* cells were transduced with pTRIPZ-HA-METTL1, TRIPZ-HA-METTL1-AFPA, or an empty vector.

Induction was achieved after 3 days of treatment with 0.1–1 µg/ml of doxycycline (Santa Cruz). Silencing or expression efficiencies were tested by RT-qPCR and Western blot.

### m^7^G methylation analysis

Total RNA was extracted using Trizol (Honeywell, 33539), followed by DNAse I Turbo (ThermoFisher Scientific), and tRNAs size-selection using mirVana (ThermoFisher Scientific). tRNAs were de-aminoacylated in 1 mM EDTA, 0.1 M Tris-HCl pH 9.0, for 30 min at 37 °C. tRNAs were used for library preparation or treated with 0.2 M Tris-HCl pH, 0.01 M MgCl_2_, 0.2 M KCl and 10 μg of m^7^GMP (Sigma) for 5 min at 85 °C [68]. 0.5 M NaBH_4_ was added and followed by 30 min ice incubation with aniline (Sigma-Aldrich). T4 PNK (NEB) treatment was followed by library preparation using NEXTFLEX Small RNA-Seq Kit v3 (Bioo Scientific Corp.). All samples were multiplexed and sequenced in HiSeq2500 (Illumina).

Raw sequencing FASTQ files were trimmed with ‘*cutadapt’* retaining reads with a minimum length of 23 nt. Reads were subsequently trimmed by 4 nt on both ends following platform recommendations. Reads were aligned to the GRCh38 (hg38) reference genome using *bowtie* (bowtie-bio.sourceforge.net) with parameters ‘-m 500 -v 2’, allowing a maximum of 500 multiple mappings and two mismatches. tRNA genomic coordinates on hg38 were obtained from the ‘*GtRNAdb’* database (gtrnadb.ucsc.edu). Reads on tRNA coordinates were processed using ‘*htseq-count’* from the Python package ‘*HTSeq*’ (htseq.readthedocs.io) with the *‘-samout’* option and counts for individual reads representing tRFs and their multiple sequence alignments on tRNAs were extracted from the resulting sam files using custom PERL scrips. Fragments with less than 10 counts over all samples were removed and a pseudo-count of 1 was added. Count data of tRFs, which were shorter than 0.7 of the full tRNA length, was normalised, and differential abundance of tRFs was evaluated for 3 replicates per condition or treatment using the R package ‘*DESeq2’* (https://bioconductor.org/packages/release/bioc/html/DESeq2.html). To determine tRNA fragmentation at m^7^G position, we applied the statistical model in ‘*DESeq2’* with *‘design* ~ *(condition + treated + condition:treated’*, where condition represented *KO* and WT).

### Mouse experimental procedures

Mice were maintained at the Animal Research Core Facility at the University of Salamanca, in ventilated filter cages under Specific Pathogen Free conditions with food and water *ad libitum*. All mouse experiments were performed following the ethical guidelines established by the Biosafety and Bioethics Committee at the University of Salamanca and by the Competent Authority of the Castilla y León Government. Conditional *Mettl1* knock out mice were generated as indicated in the supplementary information.

### Statistical analysis

GraphPad Prism 8.2 software was used. For in vitro experiments, continue and normally distribution was considered; applying unpaired one- or two-tailed Student *t*-test. For in vivo experiments with ≤10 replicates, parametric Student *t*-test was applied, while for experiments with >10 replicates unpaired Student *t*-test was applied for normal distribution and Mann Whitney test for non-normal distribution. Normality was assessed with D’Agostino & Pearson and Shapiro-Wilk tests. For comparison of multiple samples, one-way or two-way ANOVA test was applied. No statistics were applied to determine sample size. In xenograft experiments, mice sacrificed before the end of the experiment were excluded from the analysis.

More methods in supplementary information.

### Supplementary information

Supplementary Figure S1

Supplementary Figure S2

Supplementary Figure S3

Supplementary Figure S4

Supplementary Figure S5

Supplementary Figure S6

Supplementary Figure S7

Supplementary Table S1

Supplementary Table S2

Supplementary information

## Data Availability

The transcriptomic data generated in this publication have been deposited in NCBI’s Gene Expression Omnibus (GEO Series accession numbers GSE190911). The mass spectrometry proteomics data have been deposited to the ProteomeXchange Consortium via the PRIDE partner repository with the data set identifier PXD036009.

## References

[1] Frye M, Harada BT, Behm M, He C (2018). RNA modifications modulate gene expression during development. Science.

[2] Frye M, Blanco S (2016). Post-transcriptional modifications in development and stem cells. Development.

[3] Vu LP, Pickering BF, Cheng Y, Zaccara S, Nguyen D, Minuesa G (2017). The N(6)-methyladenosine (m(6)A)-forming enzyme METTL3 controls myeloid differentiation of normal hematopoietic and leukemia cells. Nat Med.

[4] Barbieri I, Tzelepis K, Pandolfini L, Shi J, Millan-Zambrano G, Robson SC (2017). Promoter-bound METTL3 maintains myeloid leukaemia by m(6)A-dependent translation control. Nature.

[5] Weng H, Huang H, Wu H, Qin X, Zhao BS, Dong L (2018). METTL14 Inhibits Hematopoietic Stem/Progenitor Differentiation and Promotes Leukemogenesis via mRNA m(6)A Modification. Cell Stem Cell.

[6] Zhang S, Zhao BS, Zhou A, Lin K, Zheng S, Lu Z (2017). m6A Demethylase ALKBH5 Maintains Tumorigenicity of Glioblastoma Stem-like Cells by Sustaining FOXM1 Expression and Cell Proliferation Program. Cancer Cell.

[7] Li Z, Weng H, Su R, Weng X, Zuo Z, Li C (2017). FTO Plays an Oncogenic Role in Acute Myeloid Leukemia as a N6-Methyladenosine RNA Demethylase. Cancer Cell.

[8] Cui Q, Shi H, Ye P, Li L, Qu Q, Sun G (2017). m6A RNA Methylation Regulates the Self-Renewal and Tumorigenesis of Glioblastoma Stem Cells. Cell Rep.

[9] Nombela P, Miguel-Lopez B, Blanco S (2021). The role of m(6)A, m(5)C and Psi RNA modifications in cancer: Novel therapeutic opportunities. Mol Cancer.

[10] Boccaletto P, Machnicka MA, Purta E, Piatkowski P, Baginski B, Wirecki TK (2018). MODOMICS: a database of RNA modification pathways. 2017 update. Nucl Acids Res.

[11] Schimmel P (2018). The emerging complexity of the tRNA world: mammalian tRNAs beyond protein synthesis. Nat Rev Mol Cell Biol.

[12] Suzuki T (2021). The expanding world of tRNA modifications and their disease relevance. Nat Rev Mol Cell Biol.

[13] Rossello-Tortella M, Llinas-Arias P, Sakaguchi Y, Miyauchi K, Davalos V, Setien F (2020). Epigenetic loss of the transfer RNA-modifying enzyme TYW2 induces ribosome frameshifts in colon cancer. Proc Natl Acad Sci USA.

[14] Rosace D, Lopez J, Blanco S (2020). Emerging roles of novel small non-coding regulatory RNAs in immunity and cancer. RNA Biol.

[15] Okamoto M, Fujiwara M, Hori M, Okada K, Yazama F, Konishi H (2014). tRNA modifying enzymes, NSUN2 and METTL1, determine sensitivity to 5-fluorouracil in HeLa cells. PLoS Genet.

[16] Rapino F, Delaunay S, Rambow F, Zhou Z, Tharun L, De Tullio P (2018). Codon-specific translation reprogramming promotes resistance to targeted therapy. Nature.

[17] Begley U, Dyavaiah M, Patil A, Rooney JP, DiRenzo D, Young CM (2007). Trm9-catalyzed tRNA modifications link translation to the DNA damage response. Mol Cell.

[18] Blanco S, Bandiera R, Popis M, Hussain S, Lombard P, Aleksic J (2016). Stem cell function and stress response are controlled by protein synthesis. Nature.

[19] Blanco S, Dietmann S, Flores JV, Hussain S, Kutter C, Humphreys P (2014). Aberrant methylation of tRNAs links cellular stress to neuro-developmental disorders. EMBO J.

[20] Lin S, Liu Q, Lelyveld VS, Choe J, Szostak JW, Gregory RI (2018). Mettl1/Wdr4-Mediated m(7)G tRNA Methylome Is Required for Normal mRNA Translation and Embryonic Stem Cell Self-Renewal and Differentiation. Mol Cell.

[21] Orellana EA, Liu Q, Yankova E, Pirouz M, De Braekeleer E, Zhang W (2021). METTL1-mediated m(7)G modification of Arg-TCT tRNA drives oncogenic transformation. Mol Cell.

[22] Dai Z, Liu H, Liao J, Huang C, Ren X, Zhu W (2021). N(7)-Methylguanosine tRNA modification enhances oncogenic mRNA translation and promotes intrahepatic cholangiocarcinoma progression. Mol Cell.

[23] Ma J, Han H, Huang Y, Yang C, Zheng S, Cai T, et al. METTL1/WDR4-mediated m(7)G tRNA modifications and m(7)G codon usage promote mRNA translation and lung cancer progression. Mol Ther. 2021; 11:e661. 10.1016/j.ymthe.2021.08.005 PMC8636169 34371184

[24] Tian QH, Zhang MF, Zeng JS, Luo RG, Wen Y, Chen J (2019). METTL1 overexpression is correlated with poor prognosis and promotes hepatocellular carcinoma via PTEN. J Mol Med (Berl).

[25] Ying X, Liu B, Yuan Z, Huang Y, Chen C, Jiang X (2021). METTL1-m(7) G-EGFR/EFEMP1 axis promotes the bladder cancer development. Clin Transl Med.

[26] Han H, Yang C, Ma J, Zhang S, Zheng S, Ling R (2022). N(7)-methylguanosine tRNA modification promotes esophageal squamous cell carcinoma tumorigenesis via the RPTOR/ULK1/autophagy axis. Nat Commun.

[27] Chen J, Li K, Chen J, Wang X, Ling R, Cheng M (2022). Aberrant translation regulated by METTL1/WDR4-mediated tRNA N7-methylguanosine modification drives head and neck squamous cell carcinoma progression. Cancer Commun (Lond).

[28] Chen Z, Zhu W, Zhu S, Sun K, Liao J, Liu H (2021). METTL1 promotes hepatocarcinogenesis via m(7) G tRNA modification-dependent translation control. Clin Transl Med.

[29] Chen B, Jiang W, Huang Y, Zhang J, Yu P, Wu L (2022). N(7)-methylguanosine tRNA modification promotes tumorigenesis and chemoresistance through WNT/beta-catenin pathway in nasopharyngeal carcinoma. Oncogene.

[30] Liu Y, Yang C, Zhao Y, Chi Q, Wang Z, Sun B (2019). Overexpressed methyltransferase-like 1 (METTL1) increased chemosensitivity of colon cancer cells to cisplatin by regulating miR-149-3p/S100A4/p53 axis. Aging (Albany NY).

[31] Marchand V, Ayadi L, Ernst FGM, Hertler J, Bourguignon-Igel V, Galvanin A (2018). AlkAniline-Seq: Profiling of m(7) G and m(3) C RNA Modifications at Single Nucleotide Resolution. Angew Chem Int Ed Engl.

[32] Lin S, Liu Q, Jiang YZ, Gregory RI (2019). Nucleotide resolution profiling of m(7)G tRNA modification by TRAC-Seq. Nat Protoc.

[33] Zueva VS, Mankin AS, Bogdanov AA, Baratova LA (1985). Specific fragmentation of tRNA and rRNA at a 7-methylguanine residue in the presence of methylated carrier RNA. Eur J Biochem.

[34] Flores JV, Cordero-Espinoza L, Oeztuerk-Winder F, Andersson-Rolf A, Selmi T, Blanco S (2017). Cytosine-5 RNA Methylation Regulates Neural Stem Cell Differentiation and Motility. Stem Cell Rep.

[35] Gkatza NA, Castro C, Harvey RF, Heiss M, Popis MC, Blanco S (2019). Cytosine-5 RNA methylation links protein synthesis to cell metabolism. PLoS Biol.

[36] Laskowska E, Kuczynska-Wisnik D, Lipinska B (2019). Proteomic analysis of protein homeostasis and aggregation. J Proteom.

[37] Kim HK, Yeom JH, Kay MA (2020). Transfer RNA-Derived Small RNAs: Another Layer of Gene Regulation and Novel Targets for Disease Therapeutics. Mol Ther.

[38] Guzzi N, Bellodi C (2021). Stressin’ and slicin’: stress-induced tRNA fragmentation codon-adapts translation to repress cell growth. EMBO J.

[39] Wu YT, Tan HL, Shui G, Bauvy C, Huang Q, Wenk MR (2010). Dual role of 3-methyladenine in modulation of autophagy via different temporal patterns of inhibition on class I and III phosphoinositide 3-kinase. J Biol Chem.

[40] Zhou W, Li J, Lu X, Liu F, An T, Xiao X (2021). Derivation and Validation of a Prognostic Model for Cancer Dependency Genes Based on CRISPR-Cas9 in Gastric Adenocarcinoma. Front Oncol.

[41] Li L, Yang Y, Wang Z, Xu C, Huang J, Li G (2021). Prognostic role of METTL1 in glioma. Cancer Cell Int.

[42] Wang C, Wang W, Han X, Du L, Li A, Huang G (2021). Methyltransferase-like 1 regulates lung adenocarcinoma A549 cell proliferation and autophagy via the AKT/mTORC1 signaling pathway. Oncol Lett.

[43] Degenhardt K, Mathew R, Beaudoin B, Bray K, Anderson D, Chen G (2006). Autophagy promotes tumor cell survival and restricts necrosis, inflammation, and tumorigenesis. Cancer Cell.

[44] Hasan A, Rizvi SF, Parveen S, Pathak N, Nazir A, Mir SS (2022). Crosstalk Between ROS and Autophagy in Tumorigenesis: Understanding the Multifaceted Paradox. Front Oncol.

[45] Rothkamm K, Barnard S, Moquet J, Ellender M, Rana Z, Burdak-Rothkamm S (2015). DNA damage foci: Meaning and significance. Environ Mol Mutagen.

[46] Cassidy LD, Narita M Autophagy at the intersection of aging, senescence, and cancer. *Mol Oncol* 2022. 10.1002/1878-0261.13269 PMC9490138 35689420

[47] Gorgoulis V, Adams PD, Alimonti A, Bennett DC, Bischof O, Bishop C (2019). Cell Senescence: Defining a Path Forw Cell.

[48] Wang L, Lankhorst L, Bernards R (2022). Exploiting senescence for the treatment of cancer. Nat Rev Cancer.

[49] Hernandez-Segura A, de Jong TV, Melov S, Guryev V, Campisi J, Demaria M (2017). Unmasking Transcriptional Heterogeneity in Senescent Cells. Curr Biol.

[50] Trotman LC, Niki M, Dotan ZA, Koutcher JA, Di Cristofano A, Xiao A (2003). Pten dose dictates cancer progression in the prostate. PLoS Biol.

[51] Choi N, Zhang B, Zhang L, Ittmann M, Xin L (2012). Adult murine prostate basal and luminal cells are self-sustained lineages that can both serve as targets for prostate cancer initiation. Cancer Cell.

[52] Wang X, Kruithof-de Julio M, Economides KD, Walker D, Yu H, Halili MV (2009). A luminal epithelial stem cell that is a cell of origin for prostate cancer. Nature.

[53] Wang ZA, Mitrofanova A, Bergren SK, Abate-Shen C, Cardiff RD, Califano A (2013). Lineage analysis of basal epithelial cells reveals their unexpected plasticity and supports a cell-of-origin model for prostate cancer heterogeneity. Nat Cell Biol.

[54] Wang ZA, Toivanen R, Bergren SK, Chambon P, Shen MM (2014). Luminal cells are favored as the cell of origin for prostate cancer. Cell Rep..

[55] Karthaus WR, Iaquinta PJ, Drost J, Gracanin A, van Boxtel R, Wongvipat J (2014). Identification of multipotent luminal progenitor cells in human prostate organoid cultures. Cell.

[56] Roviello G, Catalano M, Ottanelli C, Giorgione R, Rossi V, Gambale E (2022). Castration-resistant prostate cancer with bone metastases: toward the best therapeutic choice. Med Oncol.

[57] Lopez J, Anazco-Guenkova AM, Monteagudo-Garcia O, Blanco S. Epigenetic and Epitranscriptomic Control in Prostate Cancer. Genes (Basel) 2022;13:378. 10.3390/genes13020378 PMC8872343 35205419

[58] Yankova E, Blackaby W, Albertella M, Rak J, De Braekeleer E, Tsagkogeorga G (2021). Small-molecule inhibition of METTL3 as a strategy against myeloid leukaemia. Nature.

[59] Li J, Wang L, Hahn Q, Nowak RP, Viennet T, Orellana EA (2023). Structural basis of regulated m(7)G tRNA modification by METTL1-WDR4. Nature.

[60] Ruiz-Arroyo VM, Raj R, Babu K, Onolbaatar O, Roberts PH, Nam Y (2023). Structures and mechanisms of tRNA methylation by METTL1-WDR4. Nature.

[61] Guzzi N, Ciesla M, Ngoc PCT, Lang S, Arora S, Dimitriou M (2018). Pseudouridylation of tRNA-Derived Fragments Steers Translational Control in Stem. Cells Cell.

[62] Cosentino C, Toivonen S, Diaz Villamil E, Atta M, Ravanat JL, Demine S (2018). Pancreatic beta-cell tRNA hypomethylation and fragmentation link TRMT10A deficiency with diabetes. Nucl Acids Res.

[63] Pandey KK, Madhry D, Ravi Kumar YS, Malvankar S, Sapra L, Srivastava RK (2021). Regulatory roles of tRNA-derived RNA fragments in human pathophysiology. Mol Ther Nucl Acids.

[64] Schumacher B, Pothof J, Vijg J, Hoeijmakers JHJ (2021). The central role of DNA damage in the ageing process. Nature.

[65] Collado M, Gil J, Efeyan A, Guerra C, Schuhmacher AJ, Barradas M (2005). Tumour biology: senescence in premalignant tumours. Nature.

[66] He S, Sharpless NE (2017). Senescence in Health and Disease. Cell.

[67] Carpenter VJ, Saleh T, Gewirtz DA. Senolytics for Cancer Therapy: Is All That Glitters Really Gold? Cancers (Basel). 2021;13. 10.3390/cancers13040723 PMC7916462 33578753

[68] Zorbas C, Nicolas E, Wacheul L, Huvelle E, Heurgue-Hamard V, Lafontaine DL (2015). The human 18S rRNA base methyltransferases DIMT1L and WBSCR22-TRMT112 but not rRNA modification are required for ribosome biogenesis. Mol Biol Cell.

