# Supplementary figures and images for: N7-methylguanosine methylation of tRNAs regulates survival to stress in cancer
Source: Oncogene. 2023 Sep 2;42(43):3169–81. doi: 10.1038/s41388-023-02825-0 (PMC10589097; doi:10.1038/s41388-023-02825-0)

# Supplementary Figure S1

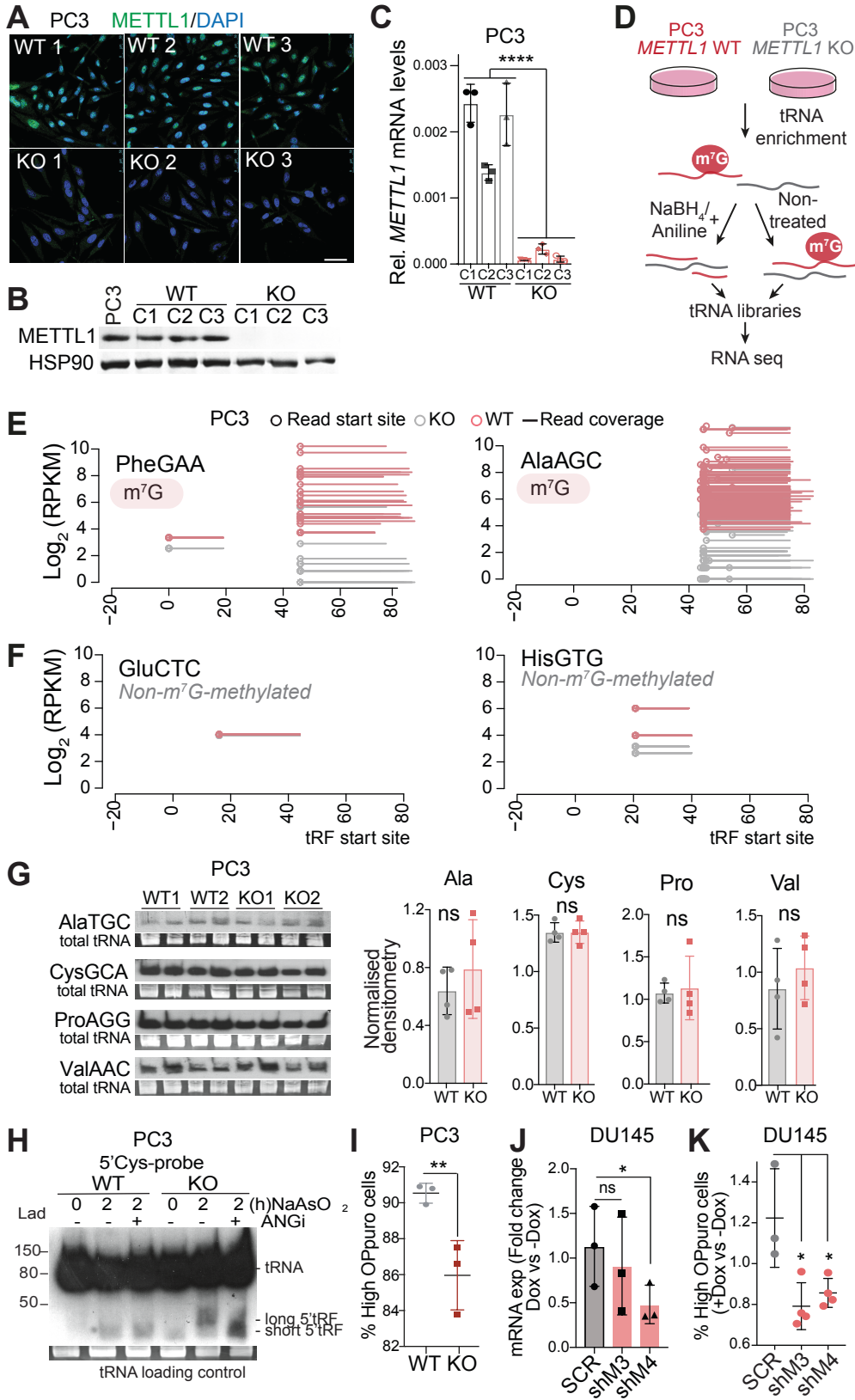

Supplement: Supplementary file 1 — Supplementary Figure S1 [file 41388_2023_2825_MOESM1_ESM.pdf]

# Supplementary Figure S2

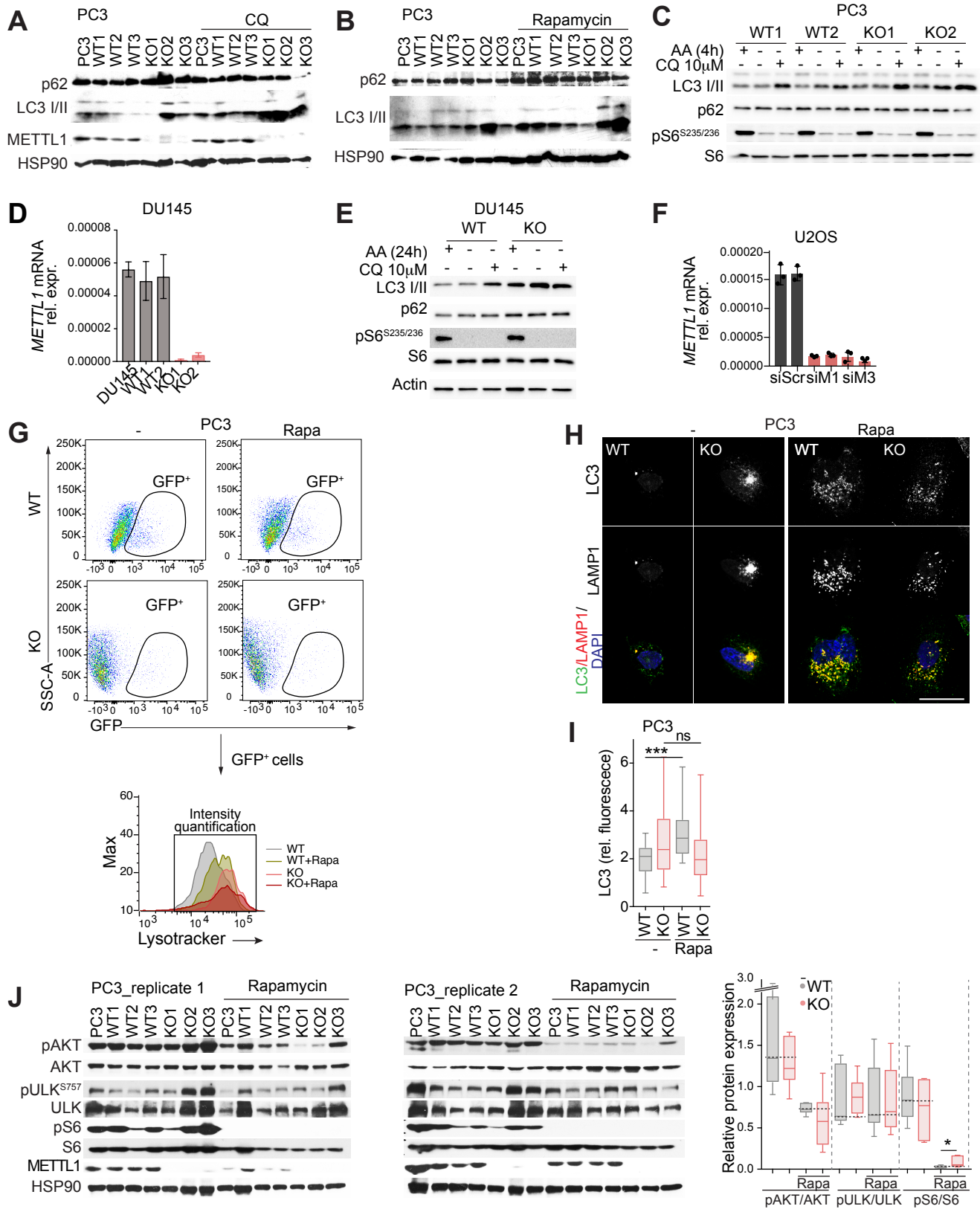

Supplement: Supplementary file 2 — Supplementary Figure S2 [file 41388_2023_2825_MOESM2_ESM.pdf]

## Supplementary Figure S3

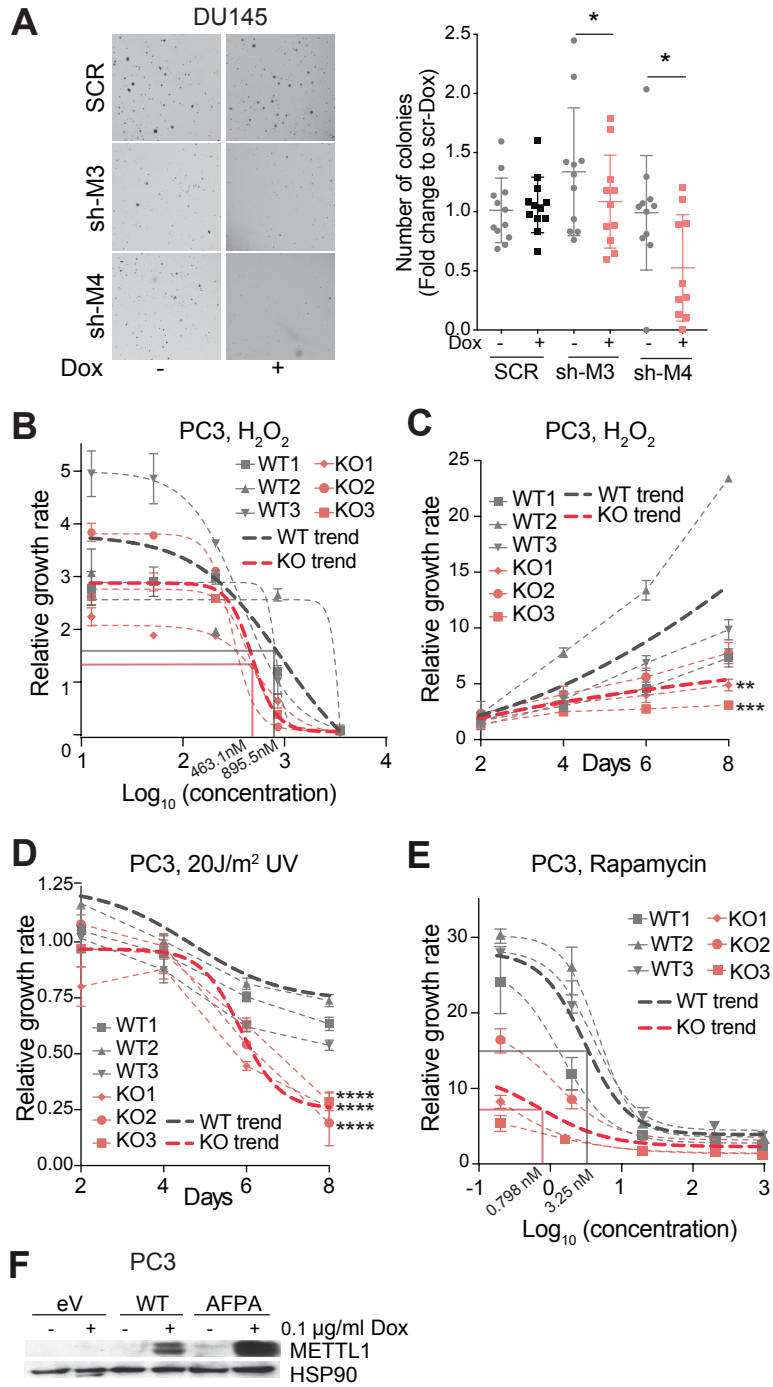

Supplement: Supplementary file 3 — Supplementary Figure S3 [file 41388_2023_2825_MOESM3_ESM.pdf]

# Supplementary Figure S4

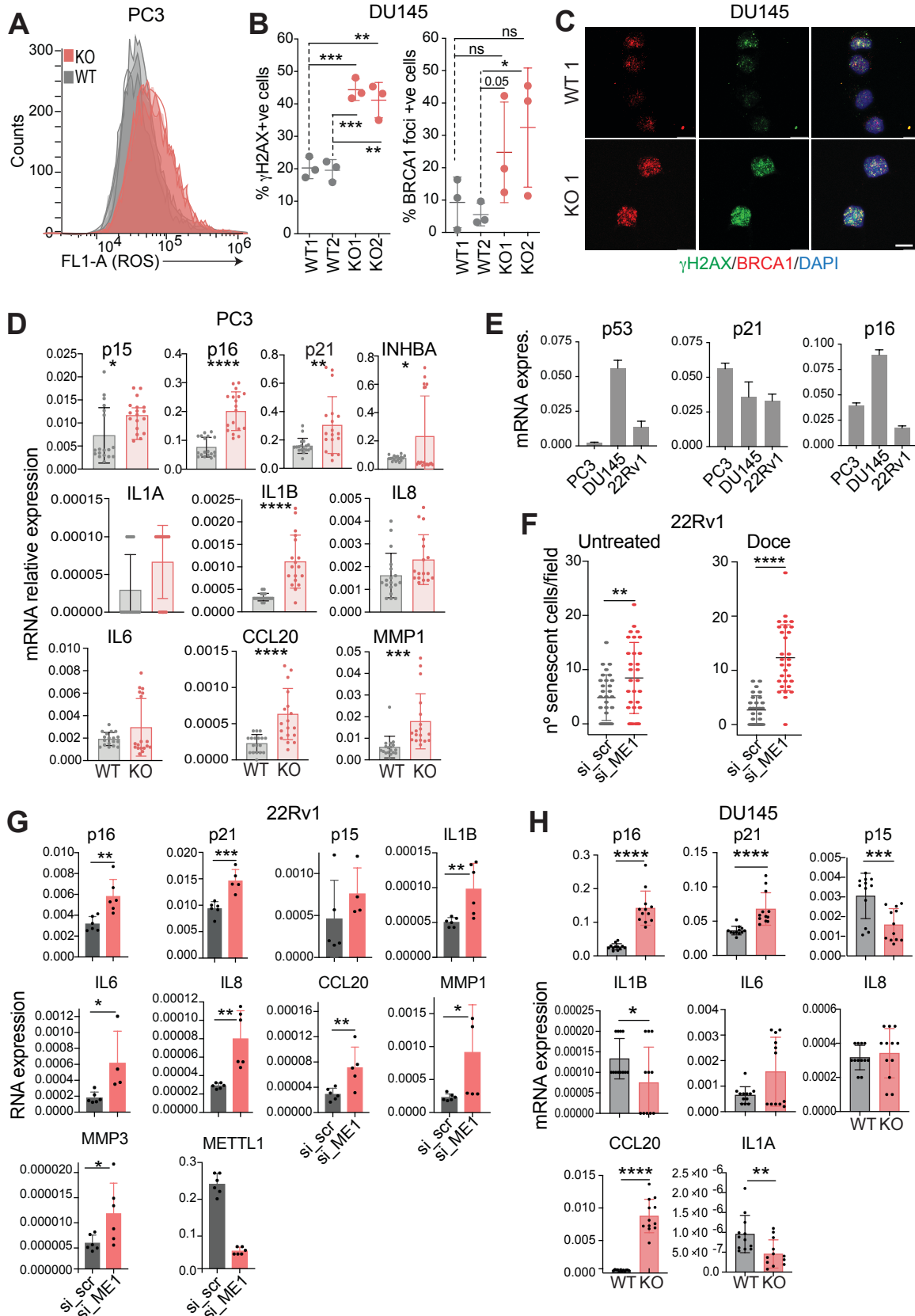

Supplement: Supplementary file 4 — Supplementary Figure S4 [file 41388_2023_2825_MOESM4_ESM.pdf]

# Supplementary Figure S5

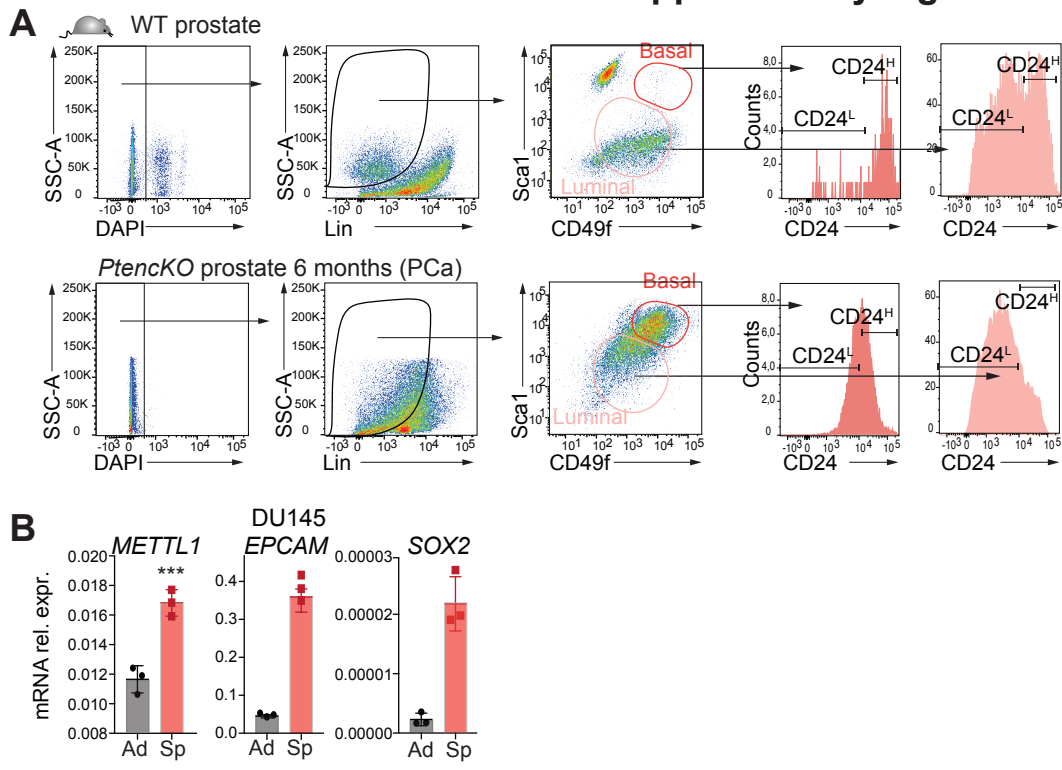

Supplement: Supplementary file 5 — Supplementary Figure S5 [file 41388_2023_2825_MOESM5_ESM.pdf]

## Supplementary Figure S6

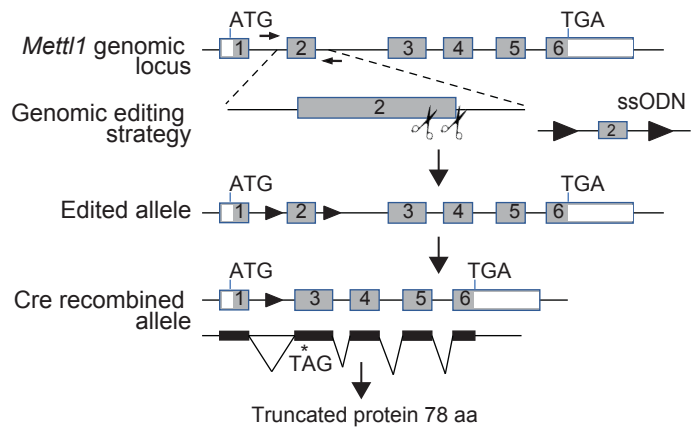

Supplement: Supplementary file 6 — Supplementary Figure S6 [file 41388_2023_2825_MOESM6_ESM.pdf]

## Supplementary Figure S7

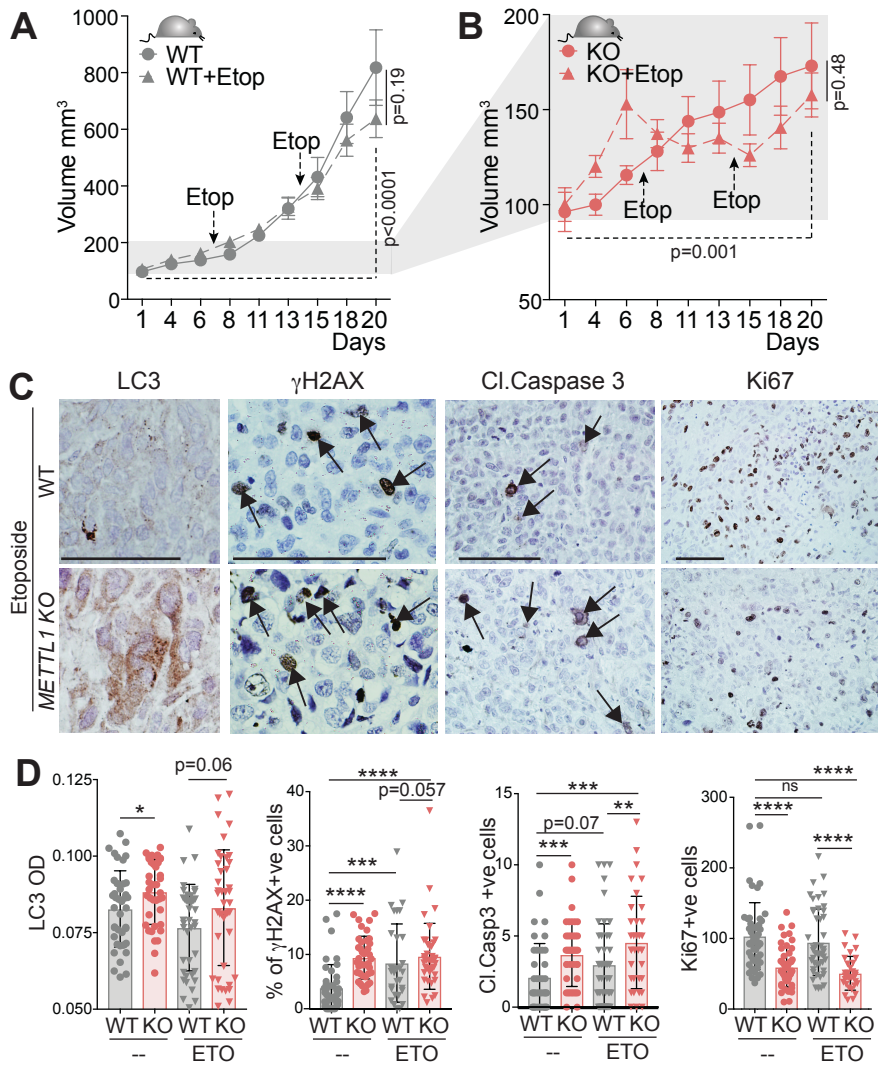

Supplement: Supplementary file 7 — Supplementary Figure S7 [file 41388_2023_2825_MOESM7_ESM.pdf]
