# Supplementary material for: N7-methylguanosine methylation of tRNAs regulates survival to stress in cancer
Source: Oncogene. 2023 Sep 2;42(43):3169–81. doi: 10.1038/s41388-023-02825-0 (PMC10589097; doi:10.1038/s41388-023-02825-0)
Supplement: Supplementary file 10 — Supplementary information [file 41388_2023_2825_MOESM10_ESM.docx]

**Supplementary information**

**Supplementary methods**

**Transfection of siRNA**

U20S-GFP-LC3 or 22Rv1 cells were transfected using INTERFERin (Polyplus) following the manufacturer’s instructions. Next, 48 hours (simple transfection) or 96 and 48 hours (double transfection) before collection, cells were transfected with 25 nM of AllStars Negative Control siRNA_scramble (Qiagen, 1027280) as control or siRNA_METTL1 (siRNA METTL1-1 (Qiagen, SI00076118) or METTL1-2 (Qiagen, SI00076132)).

**Single-cell spheroids formation**

PC3 and DU145 cell lines were grown in non-adherent conditions by pre-treating p96-well plates with 12 g/L of poly-2-hydroxyethyl methacrylate (polyHEMA, Santa Cruz Biotech.) resuspended in ethanol (Supelco). Plates were dried overnight at 65ºC and 1-1.5 cells per well were seeded by performing serial dilutions. Spheres were grown in DMEM/F12 (Gibco) medium supplemented with 1% B27 (Gibco, 17504-044), 0.02 µg/ml EGF (Gibco, PH60315), 0.004 µg/ml bFGF (ThermoFisher, 68-8785-63), 8% BSA (Nzytech, MB04602) and 1% penicillin/streptomycin. Wells with single cells were selected by microscopic observation. Spheroids were grown for 18-21 days and manually counted. At least three replicates were performed. For qPCR analysis, spheroids were collected by centrifugation and RNA was extracted from either cells growing in normal conditions, or spheroids at passage 1 (Sp1) and passage 2 (Sp2).

**Soft agar colony formation assay**

Briefly, 1.5 ml of complete DMEM medium containing 0.6% of low melting agarose (Sigma-Aldrich, 2070) was poured into 6-well plates followed by incubation for 1h at 4ºC for agar solidification. A second layer with 0.3% low melting agarose and 2.500 cells in 1.5 ml of complete medium was spread over the bottom layer. Then, plates were maintained for 15 min at 4ºC to enable agar solidification and then moved to a CO_2_ incubator. Colonies were grown for 14-21 days. For colony visualization, soft-agar plates were stained with 0.005% crystal violet in 1% methanol and scanned, and the number of colonies was quantified using ImageJ. As a seeding control, 20.000 cells were seeded in 12-wells plate, collected the following day, fixed with 4% PFA, stained with 0.1% crystal violet (Sigma-Aldrich) in 10% methanol crystal violet, and dissolved in 10% acetic acid. The number of colonies was quantified using particle counter of ImageJ software and normalised with the seeding control absorbance measurement at 595 nm.

**Northern blotting**

For stress induction, cells were treated with 200µM of NaAsO_2_ for two hours, collecting the cells 2 or 8 h after stress induction. 96 μM of angiogenin inhibitor N65828 was added together with NaAsO_2_. Total RNA was extracted from PC3 using Trizol (Honeywell, 33539), and 5 to 10 μg were run in 15% polyacrylamide 8M urea-TBE gels, and transferred to a Nylon Hybond-NX membrane (GE Healthcare) in 0.5x TBE. Membranes were cross-linked with 120 mJ/cm^2^ in UV Stratalinker 2400. Membranes were pre-hybridised at 42ºC for 1 h in ULTRAhybond buffer (ThermoFisher Scientific). Single-stranded DNA probes were radiolabelled at the 5’ end by incubating 20 pmol of the specific probe with 80 mCi of ^32^P-ATP (Perkin Elmer) and T4 PNK (NEB) for 1 h at 37ºC. Probes were purified with Oligo Clean and Concentrator columns (Zymo Research) and denatured for 5 min at 95ºC. Membranes were incubated overnight with the radiolabelled probes and then washed twice with low stringent buffer (0.1xSSC, 0.1% SDS) at 42ºC for 15 min and twice with high stringent buffer (2x SSC, 0.1% SDS). Membranes were dried and exposed overnight at -80ºC with X-ray films. The oligos used were; 5’-Ala-TGC: 5’-CCACTGAGCTACATCCCC-3’. 5’-Cys: 5’-TACCCCTGAGCTATACCCCC-3’, 5’-Pro: 5’-ATACCCCTAGACCAACGAGCC-3’, 5’-Val-AAC: 5’-ACCACTACACTACGGAAAC-3’.

**Flow cytometry**

For global protein synthesis quantification using O-propargyl-puromycin (OP-puro) labelling, cells were treated with 200µM of NaAsO_2_ for two hours, collecting the cells 2 or 8 h after stress induction. For protein labelling, cells were incubated with 20 μM of OP-puro (Medchem Source LLP) for 1h at 37ºC before acquisition. Cells were trypsinised and fixed with 1% paraformaldehyde in PBS for 15 min on ice. Cells were then washed in PBS and permeabilised in 3% FBS (Sigma-Aldrich) and 0.1% saponin (Santa Cruz Biotech.) for 5 min at room temperature. OP-puro conjugation to a fluorochrome was performed for 30 minutes by an azide-alkyne cycloaddition using the Click-iT Cell Reaction Buffer Kit (Jena Bioscience) and 5 μM of Alexa Cy5.5-azide (Jena Bioscience). Then, cells were washed twice with permeabilization buffer before acquisition. Fluorescence was acquired in Accuri C6 cytometer and protein synthesis was analysed with FlowJo software (BD Biosciences).

Quantification of intracellular Reactive Oxygen Species (ROS) was performed in PC3 WT and *METTL1* *KO* cell lines. Three replicates were used per condition. Cells were resuspended in complete medium supplemented with 20 µM of 2’7’-Dichlorofluorescin Diacetate (DCHF-DA, Calbiochem) and incubated for 30 min at 37ºC in the darkness. After centrifugation, cell pellet was resuspended in homemade Hank's buffered salt solution (HBSS) supplemented with 5% FBS and measured with BD Accuri C6 (BD biosystems) within 30 min after DCHF-DA incubation. Cell debris and doublets were discriminated, and mean fluorescence intensity was quantified using FlowJo software.

For evaluation of the lysosome-autophagosome fusion, the pH sensitive probe Lysotracker DeepRed (ThermoFisher Scientific) was used. PC3 cells were transiently transfected with pEGFP-LC3 (Addgene, #21073) using JetPEI (PolyPlus). Transfected cells were selected with 50 μg/ml of G418 for three days and all experiments were performed within a week after selection for ensuring an optimal plasmid expression. Cells were treated for 24 hours with 20 nM of Rapamycin (LC laboratories), or for 6h with 10 µM chloroquine (CQ, Sigma-Aldrich). Prior to collection, cells were incubated with 50 nM Lysotracker for 30 min at 37ºC in the darkness. Three biological replicates were acquired using BD Accuri C6 (BD Biosciences).

**Quantification of protein aggregates**

Protein aggregates were quantified in PC3 using PROTEOSTAT Aggresome detection kit (Enzo LifeSciences, ENZ-51035) following kit instructions. Cells were visualised with Leica SP5 confocal microscope.

**Proteomic analysis**

The proteome composition was analysed in DU145 cells silenced for *METTL1* using doxycycline-inducible shRNA_METTL1. Cells infected for shRNA METTL1-4 were induced for 72 hours with 0.1 μg/ml of doxycycline. Non-induced cells were used as a control. Cells were lysed in 2M thiourea, 7M Urea, 4% Chaps and 200mM DTT, and extracts were cleared by centrifugation**.** Samples were incubated for 30 min at room temperature under agitation and digested following the FASP protocol. A trypsin:protein 1:10 ratio was added and incubated overnight at 37^o^C. Samples were diluted in ammonium bicarbonate to 1.5 M urea and the same trypsin ratio, and conditions as described above were added. The resulting peptides were concentrated in a speed-vac and desalted using C18 stage tips (Millipore). Samples were analysed in a hybrid trapped ion mobility spectrometry – quadrupole time of flight mass spectrometer (timsTOF Pro with PASEF, Bruker Daltonics) coupled online either to a nano Elute (Bruker) or EvoSep ONE liquid chromatograph (EvoSep). PEAKS software was used under default settings for protein identification and quantification. Searches were carried out against a human protein entry database (Uniprot/Swissprot), with precursor and fragment tolerances of 20 ppm and 0.05 Da respectively. Proteins with one peptide at FDR<1% were considered for further analysis. Intensity data were loaded onto Perseus platform ^1^ and further processed (log_2_ transformation, imputation).

To identify specific gene subsets sharing co-occurrent functional annotations linking them to particular Gene Ontology (GO) Biological Process categories with high statistical significance, the Gene Ontology tool powered by Panther (<http://geneontology.org/>) was used. Fisher's Exact test was applied with Bonferroni correction.

**RNA extraction and quantitative real-time PCR (RT-qPCR)**

RNA from cells was extracted using Nucleospin RNA kit (Macherey-Nagel) or NZY Total RNA Isolation kit (NZYTech). Concentration was measured using NanoDrop ND-1000. For cDNA synthesis, 500 ng of RNA was reverse transcribed using Maxima H Minus cDNA Synthesis Master Mix (ThermoFisher Scientific). RT-qPCR reactions were carried out either using 2x Taqman Fast Universal PCR Master Mix (Applied Biosystems) with specific TaqMan or UPL probes, or using 2x NZY qPCR Green Master Mix (NZYtech, #MB125) with specifically designed primers. QuantStudio™ 3 or QuantStudio™ 5 Real-Time PCR Systems (Applied biosystems) were used for running the reactions. Taqman probes Hs01096146_m1 and Hs02758991_g1 were used for human *METTL1* and *GAPDH* amplification respectively. For Taqman Master Mix reactions with UPL probes, the primers for human genes used were:

F-5’-CACATTCATGTGGGCATTTC-3’ and R-5’-TGCTTGTCATGAAGTCGACAG-3’ for p16, F-5’-GAATCTCCAGGGCACCAA-3’ and R-5’-TGGCATTGCAAACTGGTC-3’ for p21, F-5’-CTCATGACCAGAAAGACCATACA-3’ and R-5’-GGACACAAGCTTAAACCCAGA-3’ for *IL8*, F-5’- TGAAATCAGGTATTCAACAGAGAAA-3’ and R-5’-CTCCCTCGCTGTTTTTATGG-3’ for *IL-1B,* F-5’-ccatgtgctggtgtgtgaa and R-5’-tgtgttttagttcaatgatgatcca for *EPCAM*, F-5’-gggggaatggaccttgtatag and R-5’-gcaaagctcctaccgtacca for *SOX2,* F-5’-CTTTCCACGACGGTGACAC-3’ and R-5’-TCAGGAAGTAGTTTCCATAGGT-3’ for p53, F-5’- CCAGGAGCCCAGCTATGAAC -3’ and R-5’-CCCAGGGAGAAGGCAACTG-3’ for *IL6,* F-5’- AGATTTGTACAGGAGTCTCCGTTG-3’ and R-5’-AAGTCCACGGGCAGACGA-3’ for *IL1A*, F-5’-GGCGAATCAGAAGCAGCAAGCAAC-3’ and R-5’-ATTGGCCAGCTGCCGTGTGAA-3’ for *CCL20*, F-5’-GCGGGGACTAGTGGAGAAG-3’ and R-5’-CTGCCCATCATCATGACCT-3’ for p15, F-5’-AAAGGGAATAAGTACTGGGC-3’ and R-5’-CAGTGTTTTCCTCAGAAAGAG-3’ for *MMP1* and F-5’-TAAAGACAGGCACTTTTGG-3’ and R-5’-GAGATGGCCAAAATGAAGAG-3’ for *MMP3,* F-5’-CGGCGCTTCTGAACGCGATC and R-5’-GCTGTTCCTGACTCGGCAAACGT for *INHBA*. The ddCt values were measured and normalised against GAPDH values. Three replicates were used. Biological replicates are indicated in each experiment.

**Western Blot**

For total cell protein extraction, cells were lysed in lysis buffer (150 mM NaCl, 40 mM Tris pH 7.6, 1% Triton X-100, 1 mM EDTA, and 1 mM MgCl_2_) supplemented with cOmPlete EDTA-free proteases inhibitor cocktail (Roche) and phosphatases inhibitors (1 mM Sodium Fluoride, 1 mM Sodium Orthovanadate and 1 mM ꞵ-glycerophosphate (Sigma-Aldrich)). Proteins were cleared by centrifugation for 10 min at 13,000 rpm. Total protein concentration was determined using Pierce BCA Protein Assay Kit (ThermoFisher Scientific). Equal amounts of protein were loaded in SDS-PAGE gels and transferred onto nitrocellulose membranes (HE Healthcare) by wet transference. Membranes were blocked with TBS-T 5% skimmed milk and primary antibodies were prepared in the blocking solution. The following primary antibodies were used for Western Blot: anti-METTL1 (Abcam, Ab157097), anti-HA (Biolegend, 901514), anti-LC3 (Cell Signaling, 12741), anti-p62 (BD biosciences, 610832), anti-ULK (Cell Signaling, 8054), anti-pULK^S757^ (Cell Signaling, 6888), anti-pS6 (Cell Signaling, 4858), anti-S6 (Santa Cruz, sc-74459), anti-pAKT (Cell Signaling, 4060), anti-AKT (Cell Signaling, 9272S), anti-GAPDH (Cell Signaling, 2118) and anti-HSP90 (Santa Cruz, sc-515081). HRP-conjugated secondary antibodies anti-mouse (Cytiva, NXA931V) or anti-rabbit (Cytiva, NA934V) were incubated for 1 hour at room temperature and signals were detected using homemade ECL (0.1M Tris-HCl pH8.5, 0.2mM coumaric acid, 1.25mM Luminol) and Fujifilm super RX (Fujifilm) films. Band intensities were quantified with ImageJ software.

For autophagy dysregulation, PC3 and DU145 cells were grown in complete DMEM supplemented with 10% FBS and treated for 24 hours with 20 nM of rapamycin (LC laboratories), or for 6h with 10 µM chloroquine (CQ, Sigma-Aldrich). In addition, starvation was induced by growing cells in EBSS + Glucose (ThermoFisher Scientific) medium without amino acids and FBS. Essential and non-essential amino acids (ThermoFisher Scientific) were added to EBSS + Glucose as +AA control.

**Cell immunofluorescence**

Cells were fixed for 10 min with 4% PFA (Panreac Applichem). Cells were permeabilised with Triton X-100/PBS (Panreac Applichem), followed by blocking with specific serum, and incubated with primary antibodies prepared in blocking solution at 4ºC overnight: anti-METTL1 (Abcam, Ab157097), anti-LC3 (Cell Signaling, 12741), anti-LAMP1 (Santa Cruz, sc-20011). Secondary antibody incubation was performed for one hour with anti-Rabbit or anti-Goat conjugated to Alexa Fluor fluorophores (ThermoFisher Scientific). Nuclei were stained with DAPI (Sigma-Aldrich, D9542). For image acquisition, Leica SP5 confocal microscope was used.

For γH2AX and BRCA1 foci visualisation, PC3 and DU145 WT and *METTL1 KO* cells were seeded on coverslips. The day after, coverslips were washed once with PBS followed by treatment with pre-extraction buffer (25 mM Tris-HCl, pH 7.5, 50 mM NaCl, 1 mM EDTA, 3 mM MgCl_2_, 300 mM sucrose and 0.2% Triton X-100) for 5 min on ice. After a wash with PBS, cells were fixed with 4% paraformaldehyde for 20 min. Following one wash with PBS, cells were blocked for 1 h with 5% FBS in PBS, and then, co-stained with the primary antibodies: anti-γH2AX (Cell Signaling, 2577L) and anti-BRCA1 (Santa Cruz Biotech., sc-6954) both at 1:500 in blocking solution overnight at 4ºC, and then co-immunostained with the secondary antibodies Alexa Fluor 488 goat anti-rabbit IgG (H+L) (Invitrogen, A11034) or Alexa Fluor 594 goat anti-mouse IgG (H+L) (Invitrogen, A11032) at 1:1000 in blocking buffer. After washing with PBS and dried with ethanol 70% and 100% washes, coverslips were mounted into glass slides using Vectashield mounting medium with DAPI (Vector Laboratories). Foci immunofluorescences were analysed for quantification using a Leica Fluorescence microscope with a HCX PL APO 63x/1.4 OIL objective. In all cases, more than 100 cells were analysed per condition and the experiments were replicated independently at least three times. Representative images shown in the figure were further taken using a LEICA confocal microscope TCS SP5 was used with a HCX PL APO lambda blue 63X/ 1.4 OIL objective.

For GFP-LC3 puncta visualisation, U2OS-GFP-LC3 cells, transfected with siRNA against human *METTL1* and growing on coverslips were fixed for 10 min with 4% of PFA. Then nuclei were stained with DAPI (Sigma-Aldrich, D9542). For image acquisition Leica SP5 confocal microscope was used. For GFP puncta quantification, more than 100 cells were analysed per condition and the experiments were replicated three times.

**Senescence Associated-β-galactosidase (SA-β-Gal) staining**

Cells growing on coverslips were treated with 10 nM Docetaxel or 60 nM Doxorubicin for one or three days. Then, cells were fixed with 0.5% glutaraldehyde (Santa Cruz Biotech.) for 10 minutes at room temperature. Cells were washed twice with 1x PBS/1 mM MgCl_2_ (pH 5.5-6) and incubated in staining solution (20x KC [100 mM K_3_FE(CN)_6_ and 100 mM K_4_Fe(CN)_6_*3H_2_O in PBS (Sigma-Aldrich)], 20x X-Gal solution (Santa Cruz Biotech.) diluted to 1x in PBS/1mM MgCl_2_ at pH 5.5-6) for 48 hours at 37°C. Coverslips were mounted in 4.8% Mowiol 4-88 (Sigma-Aldrich), 12% glycerol (VWR), 0.05 M Tris. Images were acquired with an Olympus BX-51 microscope with a digital camera Olympus DP70, and β-galactosidase positive cells were manually counted. At least 100 cells were analysed per condition and the experiments were replicated three times.

**Growth curves and survival curves**

Cells were maintained in complete medium during the experiment. Day 0 was taken as seeding control and was considered less than 24 h after seeding. When working with cells with doxycycline-inducible vectors, 0.1 µg/ml of doxycycline was added to the cells. For stress induction, 15 nM Docetaxel (LC laboratories), 3725 nM of freshly prepared H_2_O_2_ (Santa Cruz Biotech.), 20 nM Rapamycin (LC Laboratories), 20 J/cm^2^ UV pulse, 7 µM Etoposide (Sigma-Aldrich) were prepared in complete DMEM. Evaluation of 3-methyl adenine (3MA, Sigma-Aldrich) was performed in Earle’s Balance Salts Solution medium (EBSS, Sigma-Aldrich, E2888) supplemented with 4.5 g/L of glucose and 2 mM 3MA. Treatments were added 8 hours after curve seeding and medium was refreshed every two days. For collection, cells were fixed with 4% paraformaldehyde (Panreac), and stained with 0.1% crystal violet (Sigma-Aldrich) in 10% methanol for 1 hour. Then cells were washed with dH_2_O and dried overnight at room temperature. Crystal violet was dissolved in 10% acetic acid (Panreac), for 40 min and absorbance at 595 nm was measured using Plate Infinite reader 200 Pro (TECAN) to quantify cell density. All data were normalised to day 0 measurements. At least 4 replicates were performed per biological replicate.

**Prostate dissociation and sorting of normal and tumoural prostate cell populations**

Prostates of 5 months old *WT, PbCre/Pten^fl/fl^/Mettl1^+/+^* and *PbCre/Pten^fl/fl^/ Mettl1^fl/fl^* mice were dissected and dissociated for 1 hour at 37ºC in rotation with DMEM-F12 medium containing 5% FBS and 1x collagenase Type 1 (ThermoFisher) and 1x hyaluronidase (Stem Cell). After centrifugation, the pellet was incubated for 1 hour at 37ºC with 0.25% trypsin, followed by treatment with 5 mg/ml dispase II (Sigma-Aldrich) and 1 U/µl DNAse (Sigma-Aldrich) for 30 min at 37ºC. Dissociated cells were then filtered through a 0.45 µm cell strainer and stained for prostate populations with specific fluorochrome- or biotin-conjugated primary antibodies for 15-30 min in the dark. For Lineage negative: CD31-Biotin (13-0311-82, eBioscience, at 1:250), CD45-Biotin (15886978, BD Biosciences, at 1:500), Ter119-Biotin (13-5921-82, eBioscience, 1:100). And prostate specific: Sca1-APC (17-59581-82, eBioscience, 1:100), CD49f-PE (12-0495-83, eBioscience, 1:500), CD24-FITC (560992, BD Pharmingen, 1:100). For lineage negative antibodies, cells were washed and incubated with Streptavidine-eFluor710 (49-4317-82, eBioscience, 1:100) for 15 min in the dark. The cells were washed and analysed and/or sorted using BD FACS Aria II (BD Biosciences). FACS analysis was performed using FlowJo software. DAPI gating was used to separate viable from dead cells and Linage negative to separate epithelial and stromal cells from blood and endothelial cells.

RNA extraction was performed using Total RNA Purification Plus Kit (NorGen Biotek Corp, 48300). All RNA was reverse-transcribed using Maxima H Minus cDNA Synthesis Master Mix (ThermoFisher Scientific). RT-qPCR reactions were carried out either using 2x Taqman Fast Universal PCR Master Mix (Applied Biosystems) with specific UPL probes. QuantStudio™ 3 or QuantStudio™ 5 Real-Time PCR Systems (Applied biosystems) were used. For Taqman Master Mix reactions the TaqMan probes used were: for mouse GAPDH: Mm99999915_g1; for mouse CD49F: Mm01333831_m1. For UPL probes, the primers for mouse genes used were: METTL1_Fw: gctatggtggcttgttagtgg; METTL1_Rv: cttcacccgaatctccagac with probe UPL #16 (Roche); Sca1_Fw: cccctaccctgatggagtct; Sca1_Rv: tgttctttactttccttgtttgagaa with probe UPL #16; Krt5_Fw: cagagctgaggaacatgcag; Krt5_Rv: cattctcagccgtggtacg with probe #22.

**Tissue immunohistochemistry and immunofluorescence**

Mouse tissue was collected and fixed overnight with 4% paraformaldehyde, transferred to 70% EtOH, embedded in paraffin, and sectioned with 5-10 μm of thickness. A standard immunohistochemistry or immunofluorescence protocol was employed and ImmPRESS™ reagents (Vector Labs) were used for immunohistochemistry. Tissue slides were dewaxed, followed by antigen retrieval performed in the microwave using citrate buffer (pH 6) (Ki67, cleaved-Casp3, AR, K18, LC3, γH2AX) or Tris-EDTA buffer (pH 9) (METTL1, K14). For immunohistochemistry, peroxidase activity was blocked for 30 minutes with 3% hydrogen peroxide (Emsure). Then, slides were washed with PBS and permeabilised with 2% Triton X-100/PBS (Panreac), followed by blocking with specific serum. Thereafter, tissue sections were incubated overnight at 4ºC with primary antibodies prepared in blocking solution with the following antibodies: anti-Ki67 (Vector Labs, VP-K451, 1:100), anti-cleaved-Casp3 (Cell Signaling, 9661S, 1:100), anti-METTL1 (Invitrogen, PA5-54280, 1:100), anti-AR (Cell signalling, 5153, 1:100), anti-K14 (Covance, PRB-155P-100, 1:100), anti-K18 (Abcam, ab59400, 1:100), anti-LC3 (Cell Signaling, 12741, 1:100), anti-γH2AX (Cell Signaling, 2577L, 1:200). For tissue immunohistochemistry, secondary antibody incubation was performed with ImmPress HRP kit (Vector Laboratories) and the reaction was visualised with ImmPact DABSubstrate kit or Red Substrate kits (Vector Laboratories). Finally, slides were counterstained with Haematoxylin and Eosin (Millipore) and mounted with DPX (Sigma-Aldrich). For immunofluorescence, secondary antibody incubation was performed for one hour with anti-Rabbit Alexa Fluor 488, anti-Goat 488 or anti-Rabbit Alexa Fluor 594 antibodies (Invitrogen). Nuclei were stained with DAPI (Sigma-Aldrich, D9542). Vector® TrueVIEW® Autofluorescence Quenching Kit was used following the manufacturer's instructions for tissue autofluorescence reduction and slide mounting. For image acquisition, Olympus BX-51 Microscope and Leica DM6 B Microscope were used for immunohistochemistry and immunofluorescence slides, respectively.

For LC3 staining quantification, DAB and hematoxylin channels were separated using the H-DAB option from Colour Deconvolution plugin of ImageJ software. DAB intensity was measured and ODs were calculated using the following formula: OD=log(max intensity/Mean intensity); where max intensity is 255 for 8-bit images. For measuring DNA damage, the percentage of γH2AX positive cells was calculated by dividing the number of cells with γH2AX foci between the total number of nuclei in a total of 10 pictures per tumour.

**Paraffin-embedded prostate samples**

Paraffin-embedded prostate samples included in this study were provided by the Biobank Hospital Universitario Puerta de Hierro Majadahonda (HUPHM)/Instituto de Investigación Sanitaria Puerta de Hierro-Segovia de Arana (IDIPHISA) (PT17/0015/0020 in the Spanish National Biobanks Network), they were processed following standard operating procedures with the appropriate approval of the Ethics and Scientific Committees.

**Mouse lines and experimental procedures with mice**

All mice were maintained at the Animal Research Core Facility at the University of Salamanca, in ventilated filter cages under Specific Pathogen Free (SPF) conditions with food and water available *ad libitum*. All mouse experiments were performed following the ethical guidelines established by the Biosafety and Bioethics Committee at the University of Salamanca (under protocols #269, #506, #595) and by the Competent Authority of the Castilla y León Government.

*Mettl1^flox/flox^* allele for conditional deletion of *Mettl1* in mice, was generated by floxing the exon 2 of *Mettl1* (Supplementary Fig. S6A). After CRE activity, this genome edition will generate an early truncated METTL1 protein without catalytic activity. Two complementary RNA oligos were designed using the web tool breakingcas <http://bioinfogp.cnb.csic.es/tools/breakingcas/> as CRISPR RNA guides (crRNA): METTL1 guide 1 and guide 2 (crRNA1: rU rA rA rG rA rG rC rC rA rU rG rA rU rG rA rUr C rC rA rA rG rU rU rU rU rA rG rA rG rC rU rA rU rG rC rU; crRNA 2: rU rG rG rC rU rU rG rU rU rA rG rG rU rA rA rU rA rA rG rC rG rU rU rU rU rA rG rA rG rC rU rA rU rG rC rU). A single-strand oligo DNA (ssODN) contained two LoxP sequences in the same orientation flanking exon 2 and two single mutations destroying the PAM sequences was designed as a template for homologous recombination with the sequence CGGGTAAATAAAAATTTTAAAATATACATAAACAAAGTAGTGCTCCAGTGCCTTAGGCAGTCAGTCTCTGGGAAAACCTTACACACATCAGTACCCAGAAGAACTTGGCAGATCTGCAGCCATAGCCTCATGATGGGACTGATTCACACCCTAGAAGCCAATAACTTCGTATAATGTATGCTATACGAAGTTATGGGGACTTCCCATTACACCTTCTACATACCACATGGTCCCTGCATGCAGAGTTTTTTTCTAAGCCTACCACCCCACCCCCAACTCTTACACAAAATGTCTGCACTGGTATGTACAACAGTCTCATGTGTCTCATGTCTTCTTTCTAATAGCCCTGTGAAGCCAGAGGAAATGGACTGGTCTGAGCTTTACCCAGAGTTCTTTGCTCCGCTTATTCAAAATAAGAGCCATGATGATCCAAAAGATGAGAAAGAAAAGCACTCTGGGGCCCAAGTGGAGTTTGCAGACATAGGCTGTGGCTATGGTGGCTTGTTAGGTAATAAGCTCGCCCTTTTCTTGGGACAGGGAGAGGCCTGGGTTCTGCCATCTCAGCAGGTTAGAGGCAGGATTAGTTGACCTTTCCTCCTGGGACCAGACAGCAGTGACATCAGTGTGGAGAGCCTCCACCTTCCTTCTACTCTGGGTCATAACTTCGTATAATGTATGCTATACGAAGTTATTGTGGCCTTTGCCCTGGAGGAGGGAGGGGGCTGCCTCAGATGTATCTGAGAACCCCGGTTGTTCTGTCTAGCTCCAGTGGCTCCAGTCCCTCCGTGAGCCATTCCACTGCCTTGTGCTGGGTCAGTGAGCCGGGTCAGCACAAGCGTCAGAGATCAGCTCCAGTGGCCACTGATCCTGAA. The crRNAs, the ssODN and the tracrRNA (trans-activating CRISPR RNA) were produced by chemical synthesis (IDT). The crRNAs and tracrRNA were annealed to obtain the mature sgRNA. A mixture containing 30 ng/µl of recombinant Cas9 protein (IDT), 20 ng/µl each of each sgRNA, and 10 ng/µ) of ssODN were microinjected into C57BL6/J zygotes at the Transgenic Facility (NUCLEUS, University of Salamanca). All edited founders were identified by PCR amplification (Taq polymerase, NZYTech) with primers flanking the exon 2 (primer F 5’-TCTCCTGGTGTGCATGAAGAC-3’ and R 5′-AGGGAAGGTGGTGGAATCCC-3′). *Mettl1^flox/flox^* founders produced an amplicon of 957 base pairs (bp) for edited alleles with a new enzyme restriction site. PCR products were confirmed by Sanger sequencing. The founders were crossed with WT C57BL/6 J to eliminate unwanted off-targets. Heterozygous mice were crossed to give rise to edited heterozygous and homozygous. Genotyping was performed by PCR using primers F-5’-ATCTGCAGCCATAGCCTCAT-3’ and R-5’-TCTAACCTGCTGAGATGGCA-3’. *Pten^flox/flox^* and prostate epithelium-specific Cre recombinase *Pb-Cre4* were previously described^4^ and provided by Pr P.P. Pandolfi. *Mettl1^flox/flox^* mice were crossed to Pten^flox/flox^ and *Pb-Cre* mice.

For xenograft generation, 1.5x10^6^ of PC3 WT and *METTL1 KO* cells were subcutaneously injected mixed in a 1:1 proportion with Matrigel (Corning, 356234) in the two flanks of seven-week-old BALB/c Nu/Nu male mice. When tumour started to grow exponentially, 15 mg/kg of Docetaxel (LC laboratories) or 20 mg/kg of Etoposide (Sigma-Aldrich) were intraperitoneally injected weekly for three weeks in randomly selected animals. Tumour volume and mice weight were measured three times a week. Tumour volume was estimated using the formula volume= length x width2 x 0.526. After termination, tumours were dissected, weighted and snap-frozen in liquid nitrogen for RNA and protein extraction or fixed with 4% PFA for immunohistochemistry staining.

**Supplementary Figure legends**

**Supplementary Figure S1**. **tRNA fragment-mediated stress pathways in *METTL1-KO* cells.** **A, B, C)** Immunofluorescence (**A**), western blot (**B**) and qPCR analyses showing (**C**) *METTL1* loss in three independent clones of PC3-WT ad *METTL1-KO* cells. **D)** Experimental workflow followed to generate NaBH_4_/Aniline-treated tRNA libraries for sequencing. **E, F)** Read coverage of fragmented tRNAs after NaBH_4_/Aniline treatment of PC3 *METTL1-KO* (grey lines) and WT (red lines) RNAs for the indicated METTL1 tRNA substrates (**E**) and non-substrates (**F**). Circles represent the tRNA fragment (tRF) start sites. **G)** Northern blot detection and quantification of full-length mature tRNA substrates in two clones of PC3-WT and *METTL1-KO* cells. Mean ± SD, n=4. **H)** Northern blot analysis of a second replicate of Cys tRNA in PC3-WT and *METTL1-KO* cells unexposed (0h) or exposed to oxidative stress in the presence (+) or absence (-) of angiogenin inhibitor (ANGi). Red safe stains total tRNAs (**G, H**). **I**) Percentage of High OP-puro+ cells in PC3 *METTL1-KO* *METTL1-KO* cells versus WT cells. Mean ± SD, n=3. **J, K)** Fold change mRNA expression levels of *METTL1* (**J**) and fold change of the percentage of OP-puro+ cells (**K**) in *METTL1*-silenced DU145 cells using shRNAs and a control shRNA (SCR), and induced with doxycycline versus uninduced cells. Mean ± SD, n=3. Stats: one-tailed Student’s t-test (**C**, **G**, **I**, **J**, **K**). ns: non significative, *p<0.05, **p<0.01, ****p<0.0001.

**Supplementary Figure S2**. **Autophagy clearance in *METTL1-KO* cells.** **A, B)** Second replicates of western blots of LC3 I/II, p62, and METTL1 in three clones of PC3-WT and *METTL1-KO* cells growing in complete glucose, amino acid and serum medium, without or with chloroquine (CQ) (**A**) or rapamycin treatment (**B**). **C)** Western blot of autophagy markers in two clones of PC3-WT and *METTL1-KO* cells growing in medium without glucose and serum and with amino acids (+ AA), or without amino acids (- AA), or with CQ. **D, E)** *METTL1* mRNA relative expression levels (**D**) and western blot of autophagy markers in two clones of DU145 WT and *METTL1-KO* cells growing in medium without glucose, and without serum, and with amino acids (+ AA), or without amino acids (- AA), and with CQ. Mean ± SD, n=3. **F)** *METTL1* mRNA relative expression levels in U2OS cells transfected with siRNAs against *METTL1* (siM1, siM3) and control siRNA (siScr). Mean ± SD, n=3. **G)** Gating strategy followed to analyse by flow cytometry the lysotracker fluorescence intensity in PC3 LC3-GFP+ cells transiently transfected with pEGFP-LC3, without (-), or with rapamycin (Rapa) treatment. Quantification is shown in figure 2. **H, I)** Immunofluorescence (**H**) and quantification of endogenous LC3, and LAMP1 (**I**) in PC3-WT and *METTL1-KO* cells treated with rapamycin. Scale bar: 25 μm. Mean ± Max-Min, n=3. **J)** Western blots of PI3K-mTORC1 pathway markers, and METTL1 in PC3-WT and *METTL1-KO* cells treated or untreated with rapamycin. Right panel shows normalised protein level quantification of the indicated proteins. Mean ± Max-Min, n=6. Stats: one-tailed Student’s t-test (**I**). ***p<0.001.

**Supplementary Figure S3**. **Characterizing cell growth and survival in *METTL1*-deficient cells. A)** Colony formation in soft agar assay of DU145 cells stably expressing doxycycline-inducible shRNA against *METTL1* (shM3, shM4), and a shRNA scramble control (SCR). Mean ± SD, n=12. **B)** H_2_O_2_ dose-response curve of PC3-WT and *METTL1-KO* cells at 72 hours. Mean ± SD, n=4. IC_50_ of the average growth of all WT (grey) and *METTL1-KO* (red) clones are indicated as thicker dotted lines. **C)** Growth curve of three clones of PC3-WT and *METTL1-KO* cells treated with 3725 nM H_2_O_2_. Mean ± SD, n=6. Thicker dotted lines represent the average growth of all WT or *METTL1-KO* clone-derived cell lines. **D**) Growth curve of PC3 expressing and *METTL1-*depleted clones exposed to 20 J/cm^2^ of UV light. Mean ± SD, n=6. Thicker dotted lines represent the average growth of all WT or *METTL1-KO* clone-derived cell lines. **E)** Rapamycin dose-response curve of three distinct PC3-WT and *METTL1-KO* clones after eight days of treatment. Mean ± SD, n=4. The average IC_50_ value of all PC3-WT and *METTL1-KO* cells are represented as thicker dotted lines. **E)** Western blot of METTL1 in PC3 *METTL1-KO* cells stably expressing an empty vector (eV), HA-METTL1 (WT) or a METTL1 catalytic dead mutant (AFPA) treated with different doxycycline concentrations. Stats: one-tailed Student’s t-test (**A**), two-way ANOVA test (**C, D**). *p<0.05, **p<0.01, ***p<0.001, ****p<0.0001.

**Supplementary Figure S4**. **DNA damage and senescence in PCa cell lines in the absence of *METTL1*. A)** Flow cytometry histograms of intracellular ROS levels in PC3-WT and *METTL1-KO* cells. **B, C)** γH2AX- and BRCA1-foci positive cells quantification (**B**) and immunofluorescence (**C**) in two clones of DU145 WT and *METTL1-KO* cells. Mean ± SD, n=3, and 100 cells analysed per replicate. **D)** mRNA expression levels of SASP genes in PC3-WT and *METTL1-KO* cells. Mean ± SD, n=18 (**D**). **E)** mRNA expression levels of p53, p21, and p16 in different PCa cells. Mean ± SD, n=3. **F)** SA-β-gal activity (in blue) in untreated and Docetaxel-treated 22Rv1 cells transiently transfected with an siRNA against *METTL1* (si_MET1) or an scramble siRNA (si_scr) used as control. Mean ± SD, n=3, 10 images per replicate. **G)** mRNA expression levels of SASP genes in 22Rv1 cells transiently silenced with siRNAs against *METTL1* (si_MET1), and a scramble siRNA (si_scr) used as control. Mean ± SD, n=6. **G)** mRNA expression levels of SASP genes in DU145 WT and *METTL1-KO* cells. Mean ± SD, n=12. Stats: one-tailed Student’s t-test (**B, D, F-H**). *p<0.05, **p<0.01, ***p<0.001, ****p<0.0001.

**Supplementary Figure S5. Sorting strategy and expression of markers of prostate cells in mice and human normal and tumour samples. A)** Complete gating strategy followed to separate different prostate cell subpopulations from healthy (WT) and *PtencKO* mice according to Linage negative, Sca1, CD49 and CD24 expression cell surface markers. **B)** mRNA expression levels of *METTL1,* and the stem cell markers *EPCAM* and *SOX2* in DU145 cells growing in normal conditions (Ad) or in self-renewing conditions (spheroids: Sp). Mean ± SD, n=3.

**Supplementary Figure S6.** CRISPR Cas9 strategy to generate *Mettl1*-floxed allele in mice**.**

**Supplementary Figure S7**. **Treatment of WT and *METTL1-KO* xenografts treated with Etoposide. A, B)** Tumour growth of xenografted PC3-WT (**A**) and *METTL1-KO* cells (**B**) in athymic nude mice untreated or Etoposide-treated. Arrows indicate days of treatment. Mean ± SEM, n=9. **C, D)** Immunostained images (**C**) and quantification (**D**) of LC3 levels, γH2AX+, cleaved-Caspase 3+, and Ki67+ cells in xenografts. Arrows indicate positive cells (**C**). Mean ± SEM, n= 4 mice, 10 images per mouse. Scale bar: 50 μm. Stats: Mann Whitney test (**A, B, D**), ns: not significative, *p<0.05, **p<0.01, ***p<0.001, ****p<0.0001.

**Table legends**

**Supplementary Table S1.** Identification of m^7^G methylated tRNAs in PC3 cells. tRNA fragments sequences generated from NaBH_4_-Aniline treated PC3-WT and *METTL1-KO* derived RNAs.

**Supplementary Table S2.** Differentially expressed proteins in *METTL1*-silenced DU145 cells.

**References**

1. Tyanova S, Temu T, Sinitcyn P, Carlson A, Hein MY, Geiger T, et al. The Perseus computational platform for comprehensive analysis of (prote)omics data. Nat Methods 2016; 13:731-40.
